# Effective corrosion inhibition of mild steel in hydrochloric acid by newly synthesized Schiff base nano Co(ii) and Cr(iii) complexes: spectral, thermal, electrochemical and DFT (FMO, NBO) studies†

**DOI:** 10.1039/d2ra06571a

**Published:** 2022-11-14

**Authors:** Saad Melhi, Mahmoud A. Bedair, Eid H. Alosaimi, Ayman A. O. Younes, Walaa H. El-Shwiniy, Ahmed M. Abuelela

**Affiliations:** Department of Chemistry, College of Science, University of Bisha P.O. Box 511 Bisha 61922 Saudi Arabia; College of Science and Arts, University of Bisha P.O. Box 101 Al-Namas 61977 Saudi Arabia mbedair@ub.edu.sa m_bedier@yahoo.com; Department of Chemistry, Faculty of Science (Men's Campus), Al-Azhar University Nasr City 11884 Cairo Egypt m_bedier@azhar.edu.eg; Department of Chemistry, Faculty of Science, Zagazig University Zagazig 44519 Egypt

## Abstract

Two new cobalt(ii) and chromium(iii) complexes were synthesized and characterized by FT-IR, ^1^HNMR, UV, elemental analysis, TGA, conductivity, XRD, SEM, and magnetic susceptibility measurements. Structural analysis revealed a bi-dentate chelation and octahedral geometry for the synthesized complexes. The optical band gap of the Co(ii)-L and Cr(iii)-L complexes was found to be 3.00 and 3.25 eV, respectively revealing semiconducting properties. The X-ray diffraction patterns showed nano-crystalline particles for the obtained complexes. In addition, the synthesized metal complexes were examined as corrosion inhibitors for mild steel in HCl solution. The electrochemical investigations showed a maximum inhibition efficiency of 96.60% for Co(ii)-L and 95.45% for Cr(iii)-L where both complexes acted as mixed-type inhibitors. Frontier Molecular orbital (FMO) and Natural bond orbital (NBO) computations showed good tendency of the ligand to donate electrons to the metal through nitrogen atoms while the resultant complexes tended to donate electrons to mild steel more effectively through oxygen atoms and phenyl groups. A comparison between experimental and theoretical findings was considered through the discussion.

## Abstract 2

Two Co(ii) and Cr(iii) complexes were synthesized, characterized and examined as corrosion inhibitors. The electrochemical data showed high inhibition efficiencies with mixed-type behavior. FMO and NBO were considered for the computational analysis.

## Graphical abstract

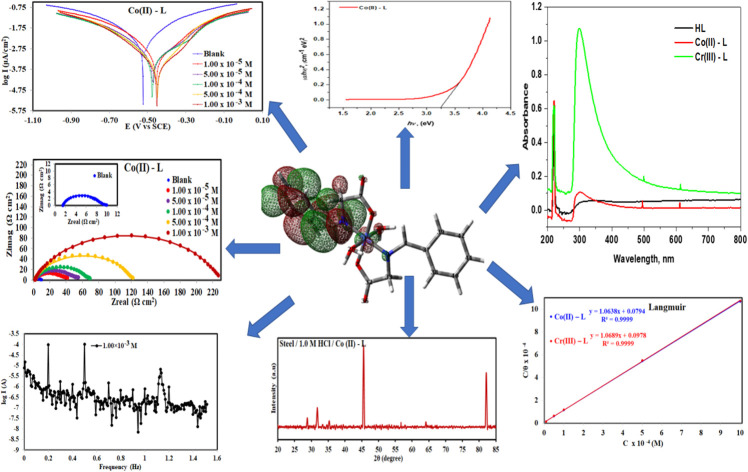

## 1. Introduction

Mild steel is a vital material with excellent mechanical properties having various applications in several industries like the petroleum and marine industries.^1^ Long-term exposure of mild steel to aggressive acids like HCl and H_2_SO_4_ acids causes severe corrosion of the metal and hence harmful destruction of steel-based equipment resulting in serious direct and indirect economic losses.^2^ The losses cost could be estimated to billions of dollars every year.^3^ Several methods and techniques were developed to resist the corrosion of metals like deposition and coating,^4–7^ however the application of corrosion inhibitors still one of the most suitable and effective methods.^8,9^ Corrosion inhibitors are characterized by high degree of adsorption ability onto metallic surface. Addition of small amounts of the inhibitor leads to fast anti-corrosion effect, therefore reducing of the corrosion rate instantly.^10,11^ Excellent corrosion inhibitor should have several active sites suitable for chemical and/or physical adsorption such as polar organic functional groups (SH, NH_2_, OH, OCH_3_, C=O, C=N), hetero organic atoms (P, S, N, O) and unsaturated aromatic π systems.^12,13^

Schiff bases are kind of molecules that can be formed by condensation of active carbonyl group with a primary amine forming, in turn, an azomethine (or known as imine) while releasing a water molecule.^14,15^ They provide flexible and supple chelation sites that have the ability to capture a wide range of transition metal ions so that numerous suitable transition-metal complexes can be obtained.^16,17^ Schiff bases as well as their metal complexes have wide applications in antibiotic, antiviral, and anticancer therapy in addition to their corrosion inhibition properties.^18–21^ They have been known as corrosion inhibitors with higher efficiency than the corresponding aldehydes or amines.^22–24^ For example, 2-hydroxy-benzoic acid [1-(2-hydroxy-phenyl)-propylidene]-hydrazide^25^ and its Mn(ii), Cu(ii) and Zn(ii) complexes, 2-amino-benzoic acid (phenyl-pyridin-2-yl-methylene)-hydrazide and its complexes with Mn(ii), Co(ii), Ni(ii), Cu(ii) and Zn(ii),^26^ a series of metal complexes of Mn(ii), Co(ii), Ni(ii), Cu(ii) and Zn(ii) with *o*-hydroxyacetophenone-2-thiophenol hydrazone^27^ and Cu(ii), Zn(ii), Mn(ii) with 2-amino-benzoic acid [1-(2-hydroxy-phenyl)-propylidene]-hydrazide^28^ were reported as highly effective corrosion inhibitors for mild steel in acidic solutions.

The current work focused on the synthesis and structural analysis of Co(ii) and Cr(iii) Schiff base-metal complexes using a variety of methods, including microchemical, physical, and spectroscopic methods (elemental analysis, UV-Vis, FTIR, ^1^H NMR, XRD, and SEM), as well as TG/DTG and kinetic parameter analysis. Our work also included the evaluation of the synthesized complexes as corrosion inhibitors using several electrochemical techniques like EFM, PDP and EIS. In addition, SEM was used to characterize the morphology of the newly synthetized complexes as well as of the mild steel samples before and after immersion in acidic solution with and without addition of such complexes. Furthermore, the charge transfer, from ligand into metal d-orbitals as well as from the metal complexes onto mild steel surface, was studied by means of DFT calculations to account for ligand–metal interactions as well as complex–metal interactions, respectively which is important to understand the ligand coordination behavior as well as the corrosion inhibition capability. Such comparison brings out significant outcomes in both the synthesis of new complex compounds and corrosion mitigation future studies.

## 2. Materials, preparation, and methods

### 2.1 Materials

The current study utilized chemicals of pure grade (hydrochloric acid 37%, benzaldehyde ≥ 99%, glycine ≥ 99%, CoCl_2_·6H_2_O ≥ 97%, CrCl_3_·6H_2_O 96%, and ethanol ≥ 99.8%) acquired from Aldrich Chemical Co (Germany). The mild steel coupons contained mainly iron with traces of C: 0.170%, Si: 0.026%, Mn: 0.460%, P: 0.012%, Cr: 0.050%, Al: 0.023%, Cu: 0.135% and Ni: 0.050%. Double-distilled water was used for preparation of 1.0 M HCl solution. The synthesized complexes were dissolved in 1.0 M HCl to prepare the following concentrations: 1 × 10^−3^ M, 5 × 10^−4^ M, 1 × 10^−4^ M, 5 × 10^−5^ M and 1 × 10^−5^ M. All glasses were immersed overnight in a chromic mixture (K_2_Cr_2_O_7_ + concentrated H_2_SO_4_), swilled entirely with bi-distilled water, and then desiccated in a 100 °C oven.

### 2.2 Preparation of the organic ligand and the metal complexes

#### 2.2.1 Fabrication of the 2-(benzylideneamino)acetic acid Schiff base (HL)

By the condensation of 0.01 mole (0.70507 g) of glycine with 0.01 mole (1.0612 g) of benzaldehyde in 50 mL of ethanol solution, Schiff base (HL) was prepared in the presence of 1 mL of glacial acetic acid using previously reported methods.^29,30^ The reaction mixture was refluxed for 8 h, cooled to 0 °C and a yellowish white precipitate was filtered off, washed several times by ethanol and dried under vacuum over CaCl_2_.

#### 2.2.2 Fabrication of the metal complexes

**Fig. 1:**
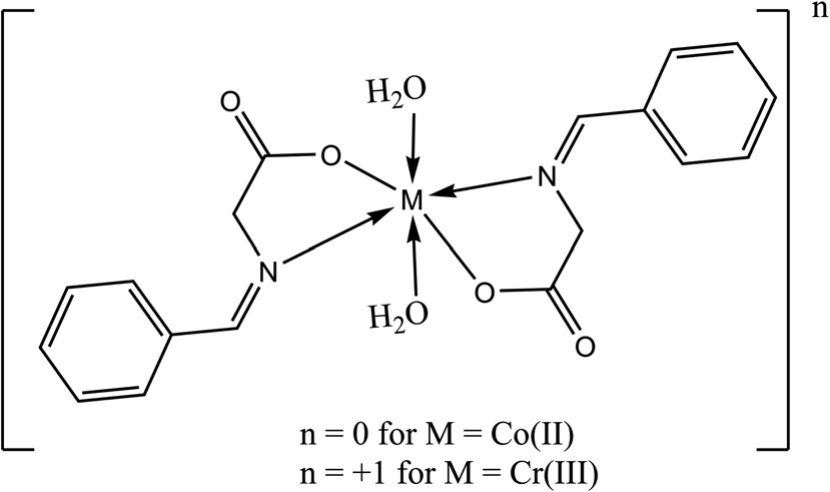
Chemical structure of the studied Schiff bases complexes.

The brown [Co(L)_2_(H_2_O)_2_]·H_2_O complex (Fig. 1) was prepared by adding 1 mmole (0.237 g) of CoCl_2_·6H_2_O in 30 mL acetone drop-wisely to a stirred solution of 2 mmole (0.326 g) of HL in 30 mL acetone and 0.080 g NaOH in 20 mL acetone. The mixture was refluxed for 6 h and the precipitate was filtered off and dried under vacuum over anhydrous CaCl_2_. The orange [Cr(L)_2_(H_2_O)_2_]Cl·3H_2_O was prepared by the same way described above using CrCl_3_·6H_2_O instead of cobalt chloride.

### 2.3 Equipment and structural characterization

The FT-IR 460 PLUS Spectrophotometer was used to capture IR spectra in Potassium bromide plates in the 4000–400 cm^−1^ range. DMSO-d6 was used as a solvent to acquire the proton nuclear magnetic resonance spectra using a Varian Mercury VX-300 NMR Spectrometer. Shimadzu UV-3101PC was used for measuring the electronic absorption spectra where ethanol was used as solvent. Shimadzu TGA-50H was used for carrying out the thermal analysis. TG-DTG measurements were performed in an environment of N_2_ at temperatures ranging from 0 °C to 1000 °C where the sample mass was precisely measured out in an aluminum crucible. Elemental analysis was carried out using a PerkinElmer 2400 CHN elemental analyzer. With a Gouy balance, a Sherwood scientific magnetic scale, and H_g_[Co(CSN)^4^] as the signing, the magnetic susceptibilities of the powdered materials were tested at ambient temperature. CONSORT K410 was used to analyze the molar conductance of 1 × 10^−3^ M solutions of the ligands and their complexes in ethanol. Each experiment was run at room temperature with freshly made solutions. The progress of the reaction was monitored by thin-layer chromatography (TLC).

### 2.4 Electrochemical measurements

EFM, EIS and PDP measurements were attained *via* a three-electrode cell consists of counter (graphite), reference (calomel) and working electrodes (mild steel of 1 cm^2^ exposed surface), using Gamry REF 3000 potentiostat/galvanostat/Zra at 30 °C. Prior to each measurement, the steel samples were immersed in the corrosive solutions for 30 min until the steady-state potential of the working electrode was achieved. The obtained data were fitted using Gamry Echem Analyst version 7.8.2. In addition, electrochemical frequency modulation was carried out using signals of two frequencies: 2 and 5 Hz, 10 mV amplitude and a base frequency of 1 Hz. Electrochemical impedance spectroscopy was performed within the frequency range of 10^−2^ and 10^5^ Hz while accompanied by an AC voltage amplitude of 10 mV. The potentiodynamic polarization was acquired within the potential range of −0.5 V and 0.5 V smearing a 1 mV s^−1^ scan rate.

In case of EFM and PDP, the inhibition efficiency was calculated by the equation:^31^1
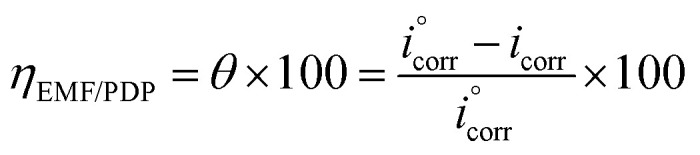
as *i*^°^_corr_ and *i*_corr_ are the current density of corrosion of steel/1.0 M HCl system without and with the investigated complexes, respectively.

In case of EIS, the efficiency was estimated from the resistance of charge transfer (*R*_ct_) as the following:^32^2
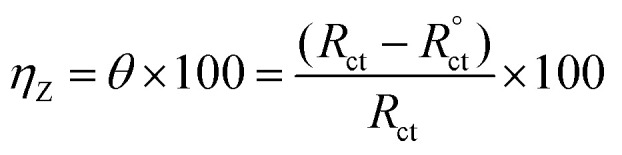
as *R*^°^_ct_ and *R*_ct_ are the resistance of charge transfer of the corrosion of steel/1.0 M HCl system without and with the investigated complexes.

### 2.5 Theoretical models

The molecular geometries of HL ligand as well as its transition-metal complexes were built, using cartesian coordinates, by Gaussview 6 (ref. 33) builder utility and were optimized according to the convergence criteria defined by Gaussian 09 software package reference^34^. The convergence was reached at maximum force of 0.1 × 10^−5^ and displacement of 1.6 × 10^−5^ within the thresholds of energy and displacement which are of 45 × 10^−5^ and 180 × 10^−5^, respectively. B3LYP DFT level of theory with 6-31g(d,p) basis set were chosen for their accuracy and affordable computational cost of analogues calculations. The frontier molecular orbitals surfaces were calculated using hirshfeld surface analysis and were represented by isovalue of 0.02 and density of 0.0004 implemented in the molecular editor of Gaussview 6 (ref. 33) to accurately approximate the exact orbital densities. The Natural bond orbitals surfaces were obtained using the NBO Version 3.1 (ref. 35) incorporated in Gaussian 09 package. The natural bond orbitals were extracted using the same edits as frontier molecular orbitals to accurately compare them.

## 3. Results and discussions

### 3.1 Structure confirmation of the prepared ligand and its complexes

The physical properties of the obtained complexes are as follow: (A) ligand; yield 98.8%, yellowish, mp: = 240 °C, MW: 163.17, (B) [Co(L)_2_(H_2_O)_2_]·H_2_O complex; yield 88.30%, brown, mp: > 360 °C, MW: 437.31, (C) [Cr(L)_2_(H_2_O)_2_]Cl·3H_2_O complex; yield 85.00%, orange, mp: > 360 °C, MW: 501.86.

#### 3.1.1 Elemental analysis, molar conductance, and magnetic moments

The isolated metal complexes were found to be only air-stable in addition to that they soluble in ethanol, dimethylformamide (DMF) and dimethyl sulfoxide (DMSO) solvents. The results of the elemental analysis are presented in Table 1 and it can be obviously concluded that the obtained chemical formulas match the proposed structures. Also, the stoichiometry of the resultant metal complexes was found to be 1 : 2/M^*n*+^ : L ratio as proposed in Fig. 1. In addition, molar conductance data can be used to conclude significant information regarding the arrangement of chelates around the central metal ions as well as the placement of anions surrounding the coordination sphere (inside, outside, or absent).^36^ The conductance of the HL ligand as well as Co(ii) and Cr(iii) complexes in DMSO (1 × 10^−3^ M) were found to be 0.0, 17.30, and 65.20 S cm^2^ mol^−1^, respectively. The Co(ii) and Cr(III) complexes' molar conductance was close with non-electrolytic and 1 : 1 electrolytes, respectively^37^. These findings were supported by a AgNO_3_ solution test for chloride anions.^38^ At ambient temperature, the effective magnetic moments (*μ*_eff_) of Co(ii) and Cr(iii) complexes were found to be 4.98 B.M. and 3.92 B.M. (paramagnetic), respectively, which is compatible with a distorted octahedral architectural configuration.^37,38^

**Table 1:** C, H, N elemental analysis of the synthesized Schiff base complexes

Complex	Molecular formula	Element%	C	H	N	Cl	Co	Cr
[Co(L)_2_(H_2_O)_2_]·H_2_O	C_18_H_22_N_2_O_7_Co	Found	49.11	5.00	6.39	—	13.40	—
		Calculated	49.44	5.07	6.41	—	13.48	—
[Cr(L)_2_(H_2_O)_2_]Cl·3H_2_O	C_18_H_26_ClN_2_O_9_Cr	Found	42.70	5.09	5.45	7.00	—	10.25
		Calculated	43.08	5.22	5.58	7.06	—	10.36

#### 3.1.2 Infrared spectra

The coordination sites involved in the chelation process were determined by comparing the IR spectra of the complexes to that of free HL ligand (Fig. S1†). Coordination has affected the intensities and wavenumbers of various peaks. The infrared spectra of Co(ii) and Cr(iii) complexes exhibit a broad band between 3375 and 3426 cm^−1^ which corresponds to the *ν*(O–H) fundamental vibrations and therefore confirms the presence of water molecules.^37^ The bands at 1650 cm^−1^ and 1570 cm^−1^ could be assigned to the stretching vibrations of *v*(C=O)_carboxyl_ and *v*(C=N)_azomethine_ of the free HL.^30,36–40^ In contrast, the absence of a band at 1650 cm^−1^ in the complexes' spectra in addition to a red shift in the characteristic wavenumber of the azomethine group to 1558 cm^−1^ for Co(ii) and 1557 cm^−1^ for Cr(iii) indicates that the *v*(C=O)_carboxyl_ and *v*(C=N)_azomethine_ groups were involved in the interaction with metal ions.^37^ A ligand having a carboxylate group can undergo coordination with metal ions in three different modes: mono-dentate, bi-dentate, and a bridge one. Moreover, the direction of an IR band shift is governed by the structure of the metal complex produced. Accordingly, while both C=O bonds are non-equivalent, in case the coordination type is mono-dentate, the anti-symmetric and symmetric (COO^−^) stretching bands would undergo blue and red shift, respectively.^29,30,41^ In the current assignment, a separation that is *ν* = *ν*_as_(COO^−^)–*ν*_*s*_(COO^−^) > 200 cm^−1^ should imply a mono-dentate bonding for the carboxylate group. Furthermore, the spectra of the isolated solid complexes reveal a series of novel bands with varying intensities that could be attributed to *ν*(M–O) and *ν*(M–N). These bands can be seen at 595 cm^−1^ and 537 cm^−1^ for Co(ii), and at 618 cm^−1^ and 598 cm^−1^ for Cr(iii) while they are absent in the HL spectra. This confirms that HL was coordinated *via* both the azomethine and carboxylic groups.

#### 3.1.3 ^1^HNMR spectra

The chemical shifts (in ppm) of the distinctive protons of the ligand HL as well as its metal complexes in DMSO-d_6_ are presented in (Fig. S2†). Due to presence of water molecules in the complexes molecules, the ^1^HNMR spectra of HL, Co(ii), and Cr(iii) complexes show O–H proton signals at 3.34 ppm, 3.37 ppm, and 3.40 ppm, respectively. Moreover, the lack of a signal owing to the –OH proton at 12.34 ppm shows that the hydroxyl group of the carboxylic group was deprotonated during complex formation.^37–40^ The complexes' ^1^HNMR spectra indicated a signal in the 8.46 ppm area which is related to the azomethine (–N=CH–) proton.^38,39^ Furthermore, the shift of the azomethine (–C=N–) carbon signal within the complex spectra relative to that of ligand spectrum, clearly suggests that the azomethine moiety was engaged in the coordination. As expected, the ligand spectrum exhibits a complicated multiplet signals in the area at 7.82 ppm due to the aromatic protons, which remains almost unchanged in the spectra of the metal complexes.

#### 3.1.4 Electronic spectra

**Fig. 2:**
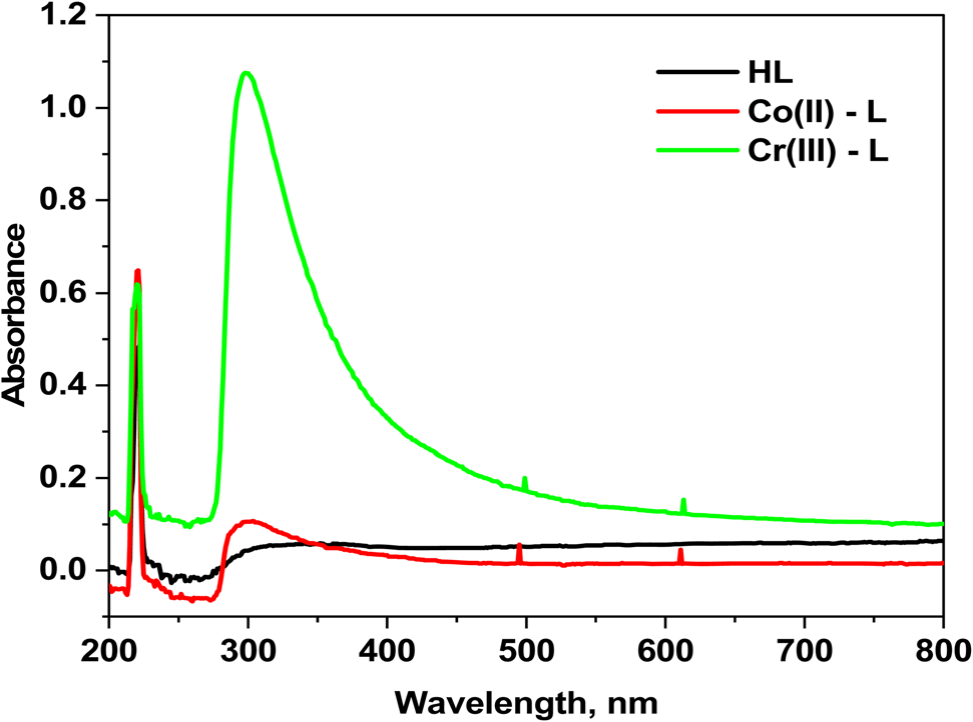
Electronic absorption spectra for HL ligand and its complexes.

The electronic spectra of HL ligand in addition to Co(ii) and Cr(iii) complexes are shown in (Fig. 2). The spectrum of HL ligand shows a prominent band at 220 nm (45 455 cm^−1^) that could be attributed to π–π* transition of (C=N)_azomethine_, however it undergoes a blue shift upon chelation, indicating that the azomethine nitrogen atom was involved in coordination. The band at 350 nm (28 571 cm^−1^) could be assigned to n–π* transition.^37^ Moreover, the [Co(L)_2_(H_2_O)_2_]Cl_2_·H_2_O complex electronic spectrum shows two wide bands at 495 nm (21 052 cm^−1^) and 611 nm (16 366 cm^−1^) due to ^4^T_1g_(F) → ^4^T_1g_(P) (*ν*_3_) and ^4^T_1g_(F) → ^4^A_2g_(F) (*ν*_2_) transitions, respectively in an octahedral high-spin d^7^ (ref. 39) (Fig. 2). The calculated values corresponding to the ligand region parameters Dq, B, and *β* are 909 cm^−1^, 957 cm^−1^, and 0.87 cm^−1^, respectively revealing that *β* is lower than unity which demonstrates the covalence of the metal–ligand bond.^39,40^ In addition, the two absorption bands at 499 and 613 nm in the electronic spectrum of the Cr(iii) complex due to ^4^A_2g_(F) → ^4^T_2g_(F) (*ν*_1_) and ^4^A_2g_(F) → ^4^T_1g_(F) (*ν*_2_) transitions, respectively beside the calculated parameters: Dq (1525 cm^−1^), B (635 cm^−1^) and *β* (0.58) prove the octahedral environment around the Cr(iii) ion.^40^

#### 3.1.5 Optical band gap energy

Tauc plot eqn (3) was used to calculate the optical band gap energy (*E*_g_) from the absorption spectra.

**Fig. 3:**
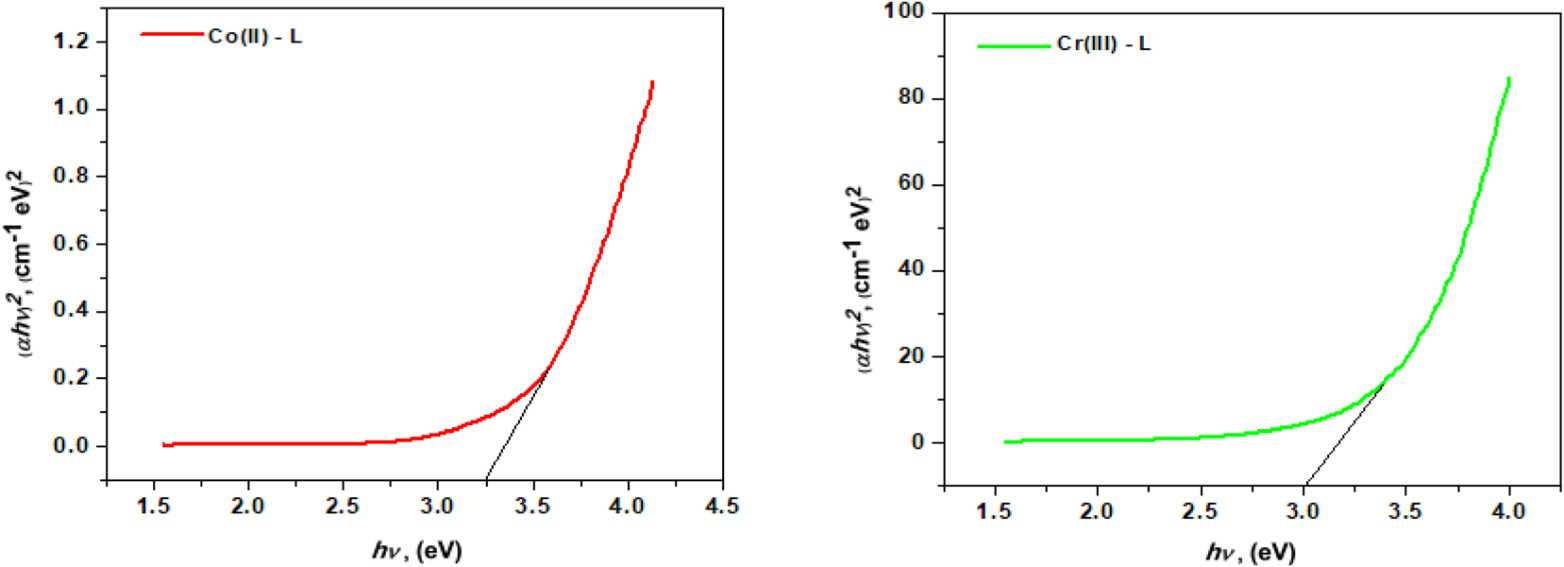
The allowed direct band gaps of the complexes.

3 (*αhv*)^*n*^ = *A* (*hv*−*E*_g_)

where *hv* is the photon energy, *h* is Planck's constant, *α* is the absorption coefficient, *E*_g_ is the optical energy gap, *A* is constant, *n* is equal to 1/2 and 2 for direct and indirect transitions, respectively. In addition, the absorption coefficient (*α*) was calculated from eqn (4):4
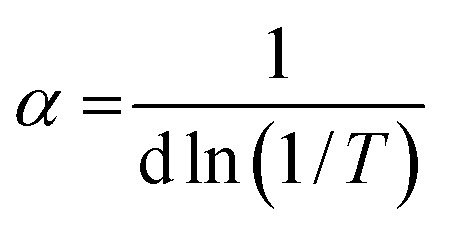
where *d* is the width of the cell and *T* is the measured transmittance. The plot of (*αhv*)^2^*vs. hv* gave a direct band gap (*E*_g_) as shown in Fig. 3. The *E*_g_ values were estimated to be 3.25 eV and 3.00 eV for Co(ii) and Cr(iii) complexes, respectively. The narrow band gap could be explained in terms of electron reallocation from ligand into metal shell so that the localized energy levels are expanded and the hence the band gap gets smaller. This result shows that the synthesized complexes could be nominated for many applications in optics, electronics and energy conversion devices.^39^ In fact small band gap facilitates electronic transitions across the HOMO–LUMO energy levels and makes the molecule more electro-conductive.^40^ It is worth to note that, from the optical properties, the synthesized compounds lie in the same range of highly efficient photovoltaic materials and could be used as semiconductors.

#### 3.1.6 Thermal analysis

**Fig. 4:**
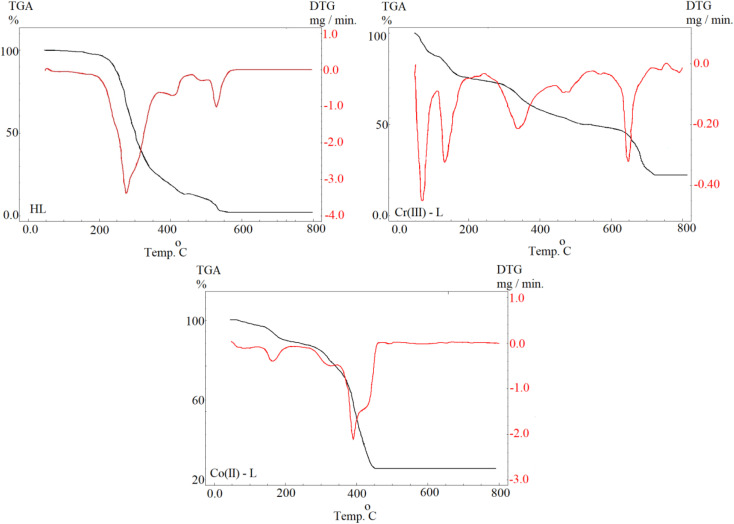
Ligand (HL) TGA curves and its complexes.

The thermal characteristics of the ligand (HL) and its complexes were determined by thermogravimetric analysis (TGA) (Fig. 4). Table 2 shows the temperature intervals and the fraction of the masses lost by the ligand and its complexes. The ligand HL was decomposed in three main stages over a temperature range of 25–800 °C. The first step occurred at 276 °C with a mass loss of (calcd = 63.74%) due to the removal of 4C_2_H_2_. The second step occurred at 410 °C with a mass loss of (calcd 26.96%) due to the loss of CO_2_. The last one, within the range 525–530 °C, was accompanied by mass loss of (calcd = 9.20%) due to the removal of ½H_2_ + ½N_2_. The thermal decomposition of [Cr(L)_2_(H_2_O)_2_]Cl_3_·3H_2_O complex occurred in four steps. At 70 °C, the first step loss was 9.24 percent (calcd 9.42 percent) which corresponds to separation of three water molecules. The second and third stages happened between 133 and 337 °C and included the loss of two coordinated water molecules in addition to three chloride anions with an estimated mass loss of 6.16 and 18.44 percent (calcd 6.28 and 18.59 percent). The last phase demonstrated the elimination of 4C_2_H_2_ + 2C_2_H_4_ + NO + NO_2_ moieties, leaving CrO + 6C as a 24.36 percent residue (calcd 24.45 percent). The TG curve of the [Co(L)_2_(H_2_O)_2_]Cl_2_·H_2_O complex indicates that it was decomposed into four phases. The initial weight loss stage, at 86 °C, had weight loss of 3.44 percent (calcd 3.54 percent) which equates to one water molecule lost. The second and third weight loss stages at decomposition temperatures of 165 °C and 320 °C, respectively showed the loss of two coordinated water molecules and chloride ions from the outer coordination sphere with weight losses of 7.00 percent and 13.86 percent (calcd 7.08 percent and 13.86 percent), respectively. The fourth weight loss stage revealed a constant weight loss between 319 and 403 °C corresponding to the loss of whole ligand molecule and 50.55 percent of the original sample with a computed weight percentage of 50.75 percent where the final product was CoO + 4C.

**Table 2:** The maximum temperature *T*_max_( °C) and weight loss values of the decomposition stages for HL and its metal complexes

Compounds (MW, MF)	*T*_max_( °C)	Weight loss (%)		Lost species
		Calc.	Found	
HL (163.17, C_9_H_9_NO_2_)	276	63.74	63.85	Decomposition of the organic ligand (loss of 4C_2_H_2_)
	410	26.97	26.96	Decomposition of the organic ligand (loss of CO_2_)
	530	9.20	9.19	Further dissociation of the organic ligand (½H_2_ + ½N_2_)
[Cr(L)_2_(H_2_O)_2_]Cl·3H_2_O(501.86, C_18_H_26_ClN_2_O_9_Cr)	70	10.75	10.24	Loss of hydrated 3H_2_O molecule
	133	7.17	7.16	Loss of coordinated 2H_2_O
	337	7.07	7.00	Loss of chloride anions
	473, 647	47.12	46.95	Dissociation of the organic ligand (4C_2_H_2_ + 2C_2_H_4_ + NO + NO_2_)
		27.89	27.36	Formation of CrO + 6C as final product
[Co(L)_2_(H_2_O)_2_]·H_2_O(437.31; C_18_H_22_N_2_O_7_Co)	86	4.11	3.98	Loss of hydrated H_2_O molecule
	165	8.23	8.10	Loss of coordinated 2H_2_O
	320	17.83	17.68	Decomposition of the organic ligand ((3C_2_H_2_)
	391	41.47	41.35	Further decomposition of the organic ligand (3C_2_H_2_ + C_2_H_4_ + NO + NO_2_) formation of CoO + 4C as final product
		28.35	28.10	

### 3.2 Electrochemical measurements

#### 3.2.1 Open circuit potential (OCP)

**Fig. 5:**
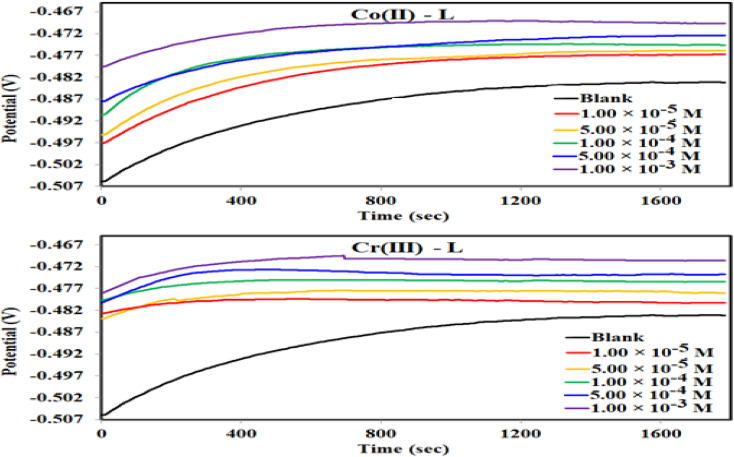
OCP–time curves for MS in 1.0 M HCl solution without and with different concentrations of Co(ii)-L and Cr(iii)-L complexes at 30 °C.

For electrochemical measurements of a corrosion reaction, the evaluation of *E*_oc_ responses *vs.* time is usually an input step which is performed to ensure attainment of a steady state. We can notice from Fig. 5 that *E*_oc_ was slightly increasing over time then became almost constant after 30 min, for the blank solution and the solutions of the metal complexes of all concentrations, as an indication that *E*_oc_ reached equilibrium. Also, in case of using Co(ii)-L and Cr(iii)-L complexes, *E*_oc_ was balanced at higher values than those of the blank solution indicating the dependance of *E*_oc_ on the presence of the inhibitors. Moreover, *E*_oc_ shifts to more positive values as the concentration of the inhibitors increased. This shift in the more positive direction (more noble) is due to the adsorption process of the complexes on steel surface and blocking of the active sites (anodic and cathodic sites) of corrosion.^42^ Moreover, the positive gap between *E*_ocp_ value in case of the complex containing solution *vs.* the blank is less than 85 mV for all concentrations, therefore the inhibition process includes both cathodic and anodic reactions (mixed type mechanism).^43^

#### 3.2.2 Potentiodynamic polarization (PDP)

**Fig. 6:**
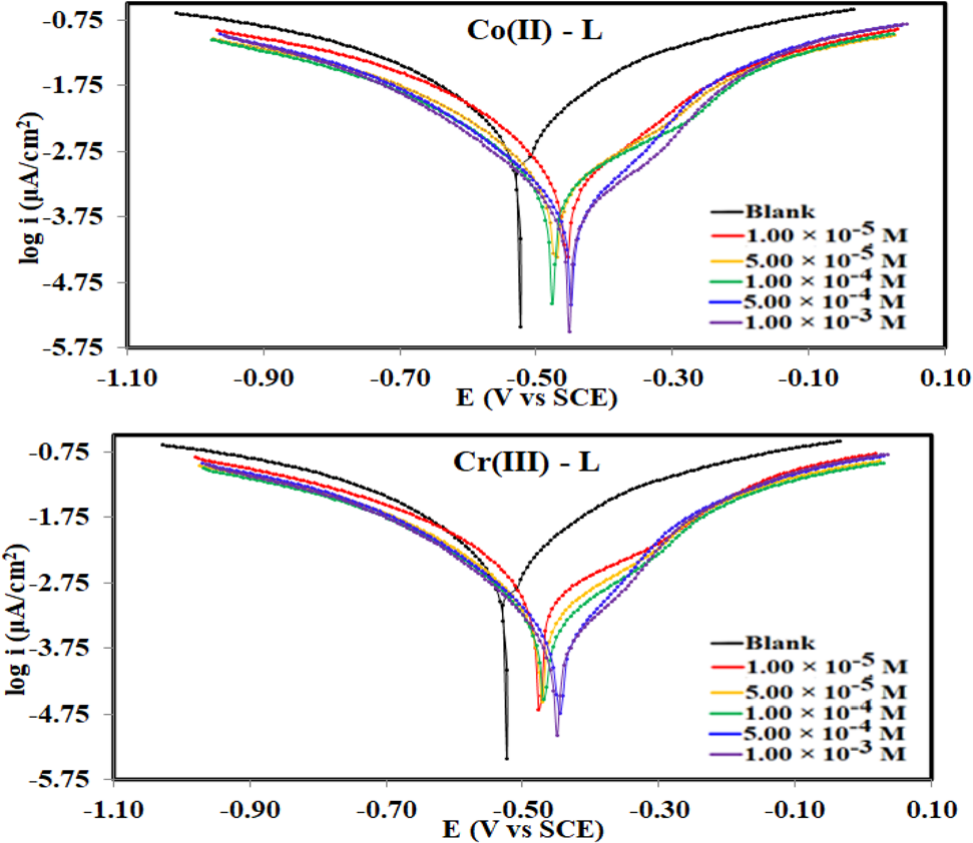
Potetiodynamic polarization curves for MS in 1.0 M HCl solution without and with different concentrations of Co(ii)-L and Cr(iii)-L complexes at 30 °C.

Fig. 6 collects the potentiodynamic polarization curves for steel, in the corrosive 1.0 M HCl solution (blank) *vs.* solutions of Co(ii)-L and Cr(iii)-L complexes, at 30 °C. The main fitting parameters from the extrapolation of the Tafel plots such as corrosion current density (*i*_corr_), corrosion potential (*E*_corr_), cathodic and anodic Tafel slope (*β*_c_ and *β*_a_) in addition to the inhibition efficiency *η*_PDP_(%) are collected in Table 3. Fig. 6 clearly shows that both the cathodic and anodic plots of the complexes solutions have been significantly shifted to lower current density areas with respect to the blank solution. This is essentially due to the binding arises between the Co(ii)-L and Cr(iii)-L molecules on the steel surface presumably *via* numerous adsorption actives sites (sources for electron donation).^44^ This finding confirms that the adsorption of the complexes molecules acts well for both cathodic and anodic sites on steel surface. As a consequence, both the hydrogen evolution reduction reaction as well as the iron dissolution oxidation reaction were retarded in the presence of the complexes which again confirms that they are mixed-type inhibitors.^45^ Data in Table 3 shows that *i*_corr_ was diminished by the addition of metal complexes which is a good indication of the inhibitor's high protection capability. Moreover, solutions with the highest concentration of Co(ii)-L and Cr(iii)-L showed *i*_corr_ of 380 μA cm^−2^ and 512 μA cm^−2^, respectively which proves the formation of a protection layer on the MS and therefore reduces the corrosion reactive sites on the investigated surface. Furthermore, the addition of Co(ii)-L and Cr(iii)-L to the acidic aggressive solution caused a +75 mV and +77 mV shift in E_corr_ with respect to that in the blank which confirms the mixed type adsorption nature of Co(ii)-L and Cr(iii)-L molecules.^46,47^ The Tafel constants (*β*_c_ and *β*_a_) were affected by the addition of both complexes indicating the interaction of the complexes with the anodic and the cathodic mechanisms elements. According to the polarization data, the protection ability approached its maximum values at 95.59% for Co(ii)-L and 94.06% Cr(iii)-L. Consequently, both Co(ii)-L and Cr(iii)-L have excellent inhibition efficiencies.

**Table 3:** Electrochemical parameters^a^ for steel dissolution in 1.0 M HCl solution containing different concentrations of the (Co(ii)-L, Cr(iii)-L) inhibitors obtained from polarization measurements at 30 °C

Inhibitor name	Conc. (M)	*E*_corr_ *vs*.SCE (mV)	*i*_corr_ (μA cm^−2^)	*β*_a_ (mV dec^−1^)	*β*_c_ (mV dec^−1^)	CR (mpy)	Δ*E*_corr_ (mV)	*θ*	*η*_PDP_%
Blank	—	−523	8624	305	333	4137	—	—	—
Co(ii)-L	1.00 × 10^−5^	−455	1480	232.7	242.9	575.7	68	0.828	82.83
	5.00 × 10^−5^	−471	1130	235.9	240.5	516.3	52	0.869	86.90
	1.00 × 10^−4^	−477	805	220.9	223.7	367.9	46	0.907	90.67
	5.00 × 10^−4^	−448	533	180.9	206.8	243.4	75	0.938	93.82
	1.00 × 10^−3^	−452	380	175	191.9	173.7	71	0.956	95.59
Cr(iii)-L	1.00 × 10^−5^	−476	1570	229.5	234.6	716.4	47	0.818	81.79
	5.00 × 10^−5^	−471	1220	228.1	235.2	557.9	52	0.859	85.85
	1.00 × 10^−4^	−467	888	216	224.9	405.8	56	0.897	89.70
	5.00 × 10^−4^	−446	661	183.8	213.9	302.2	77	0.923	92.33
	1.00 × 10^−3^	−450	512	177.6	202.9	234	73	0.941	94.06

a *E*_corr_, is the corrosion potential; *I*_corr_, is the corrosion current density: *β*_a_ and *β*_c_ are Tafel constants for both anode and cathode; *k*, is the corrosion rate; *θ*, is the surface coverage; *η*_PDP_, is the inhibition efficiency.

#### 3.2.3 Electrochemical frequency modulations (EFM)

**Fig. 7:**
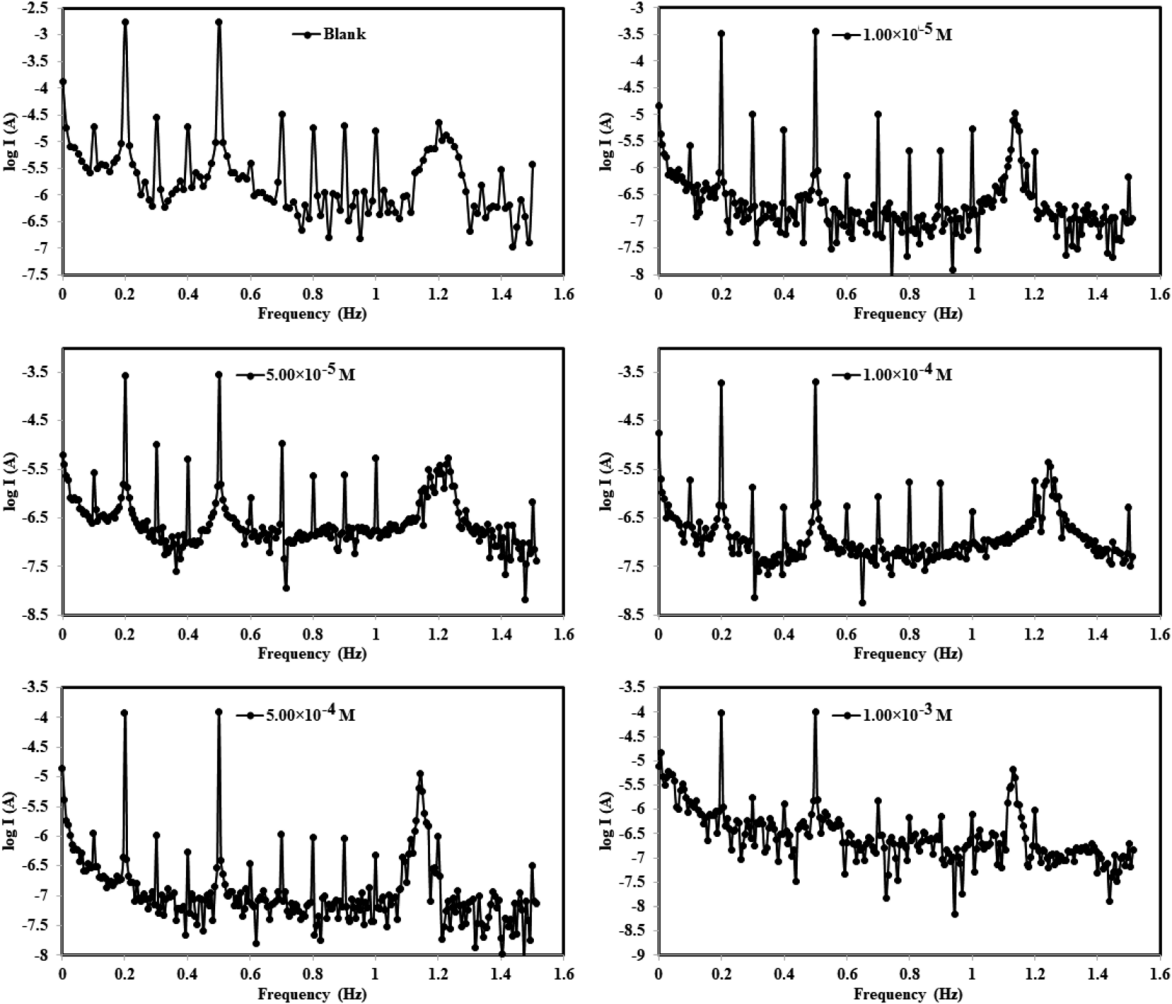
Electrochemical frequency modulation curves for MS in 1.0 M HCl solution without and with different concentrations of Co(ii)-L and Cr(iii)-L complexes at 30 °C.

The intermodulation frequency spectra for mild steel electrode dipped in 1.0 M HCl solution in absence/presence of different concentrations of Co(ii)-L complex molecules at 30 °C are presented in Fig. 7 (similar spectra were obtained for Cr(iii)-L and are available upon request). These frequency modulations were obtained by applying of 2 and 5 Hz input excitation frequencies. It is worthy to mention that two casualty factors (CF-2 and CF-3) were determined for all harmonic frequencies in addition to selected intermodulation frequencies which validate the experimental outcomes.^48^ All EFM spectra have two prominent peaks because of the designated excitation frequencies (2 and 5 Hz) as seen in Fig. 7 while peaks with weaker signals are due to the harmonic difference of certain excitation frequencies.^49^Fig. 7 reveals that the EFMs of the complexes lie in the high negative current regions relative to the blank corrosive medium, suggesting that the complex molecules reduce the flow of electrical current between the electrodes.

Moreover, the EFM parameters were obtained by fitting the intermodulation spectra and were listed in Table 4. The theoretical casualty factors CF-2 and CF-3 which are 2 and 3, respectively were determined automatically utilizing the 2 notable peaks. Table 4 shows an agreement between the estimated CF values and the theoretical ones indicating that the EFM observations are reliable.^50^ Upon the addition of the complex molecules to the corrosive solution, the values of *β*_a_ and *β*_c_ were altered. This fluctuation occurred due to that the highly electron-rich sites of molecule created an electrochemical layer on both anodic and cathodic corrosion sites. The *i*_corr_ was gradually dropped down while raising the concentrations of Co(ii)-L and Cr(iii)-L molecules until it reached 180.0 and 196.5 μA cm^−2^, respectively for the highest concentrations. The *η*_*EFM*_ was estimated from *i*_corr_ (eqn (1)) and it was found to step up from 69.54 to 93.55% with increasing complex concentrations from 1.00 × 10^−5^ to 1.00 × 10^−3^ M. The optimal percentage of protection was found to be 93.55% and 92.96% for 1.00 × 10^−3^ M of Co(ii)-L and Cr(iii)-L complex molecules, respectively at 30 °C. Consequently, the two prepared complex molecules have promising and outstanding protective performance for steel in 1.0 M HCl.

**Table 4:** Electrochemical kinetic parameters^a^ obtained by EFM technique for steel in the absence and presence of various concentrations of (Co(ii)-L, Cr(iii)-L) inhibitors in 1.0 M HCl at 30 °C

Inhibitor name	Conc. (M)	I_corr_ (μA cm−2)	*β*_a_ (mV dec^−1^)	*β*_c_ (mV dec^−1^)	CF-2	CF-3	*K* (mpy)	*θ*	*η*_EFM_%
Blank	—	2791.0	100.4	113.1	1.763	3.155	1275.0	—	—
Co(ii)-L	1.00 × 10^−5^	776.7	129.6	170.1	1.949	3.152	354.9	0.722	72.17
	5.00 × 10^−5^	529.3	109.1	145.9	1.975	3.371	241.9	0.810	81.04
	1.00 × 10^−4^	367.4	119.7	124.7	2.288	3.260	167.9	0.868	86.84
	5.00 × 10^−4^	238.2	122.8	131.5	2.097	3.063	108.8	0.915	91.47
	1.00 × 10^−3^	180.0	112.3	126.4	1.519	3.707	82.3	0.936	93.55
Cr(iii)-L	1.00 × 10^−5^	850.0	128.4	177.5	1.987	2.901	388.4	0.695	69.54
	5.00 × 10^−5^	615.9	116.9	160.5	1.962	3.023	281.4	0.779	77.93
	1.00 × 10^−4^	441.4	88.9	114.0	1.972	3.349	201.7	0.842	84.18
	5.00 × 10^−4^	256.9	98.3	113.8	2.114	3.929	117.4	0.908	90.80
	1.00 × 10^−3^	196.5	118.9	133.4	2.095	2.920	89.8	0.930	92.96

a *E*_corr_, is the corrosion potential; *I*_corr_, is the corrosion current density: *β*_a_ and *β*_c_ are Tafel constants for both anode and cathode; *k*, is the corrosion rate; *θ*, is the surface coverage; *η*_EFM_, is the inhibition efficiency.

#### 3.2.4 Electrochemical impedance spectroscopy (EIS)

**Fig. 8:**
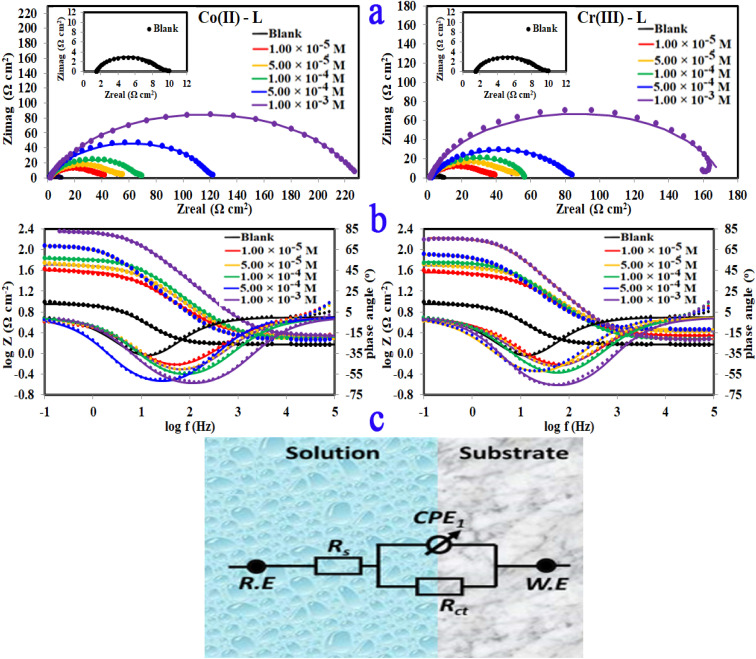
(a)Nyquist plots, (b) bode, phase angle plots and (c) equivalent circuit for MS in 1.0 M HCl solution without and with different concentrations of Co(ii)-L and Cr(iii)-L complexes at 30 °C.

The corrosion and interface conditions in 1.0 M HCl solution with and without the presence of different concentrations of the investigated complexes were studied using electrochemical impedance spectroscopy (EIS) at 30 °C. The extracted data for Nyquist and Bode plots are plotted in Fig. 8 For all different set of concentrations of Co(ii)-L and Cr(iii)-L molecules. The Nyquist graphs (Fig. 8a) show a capacitive loop, suggesting that the steel specimen in 1.0 M HCl seems to have a capacitive behavior and consequently its corrosion is governed by charge transfer process.^50^ The inhomogeneity of the examined steel surface during the EIS experiments is frequently linked to the depressed semi-circle shape of the Nyquist plots.^51^ The presence of Co(ii)-L or Cr(iii)-L molecules haven't impacted the semi-circle shapes, however the width of the semicircles was enlarged as the complexes concentration increased, implying that the corrosion reaction mechanism wasn't modified and therefore, the adsorption of the complex molecules must be occurred on the steel surface and evidently the adsorbed film thickness was gradually increased by increasing of the complexes concentration. The EIS results were fitted with a suitable equivalent circuit utilizing Echem Analyst software to determine the most suitable circuit elements that aligns the experimental EIS results. Furthermore, the chi-squared was used to assess the accuracy of the simulated data. The obtained tiny chi-squared of 10^−3^, of all samples, show that the simulated data is significantly related to the actual data.^52^Fig. 8c shows the simulated circuit (R(CPE-R)) which comprises of; (i) two resistances: one related to the solution (*R*_s_) and the other to the charge transfer (*R*_ct_), and (ii) one constant phase element (CPE). CPE is usually used instead of a double-layer capacitance (*C*_dl_) to provide an accurate and precise fit. Table 5 lists the EIS parameters obtained by fitting the experimental EIS graphs to the chosen equivalent circuit. *C*_dl_ is related to the CPE constants (*Y*_o_ and *n*) by the equation:^53^

5 *C*_dl_ = [*Y R*^1−*n*^_ct_]^1/*n*^

**Table 5:** EIS parameters^a^ for corrosion of steel in 1.0 M HCl in the absence and presence of different concentrations of (Co(ii)-L, Cr(iii)-L) inhibitors at 30 °C

Inhibitor	Conc. (M)	Rs (Ω cm^2^)	*R*_ct_ (Ω cm^2^)	*Y*o (μ Ω−1 sn cm−2)	*n*	C_dl_ (μF cm−2)	chi squared	S	α°	*τ* (s)	*θ*	*η*_z_ %
Blank	—	1.501	7.68	4109	0.852	2254.5	1.10 × 10^−3^	−0.179	−37.02	0.017	—	—
Co(ii)-L	1.00 × 10−5	2.187	37.97	1398	0.756	542.0	5.02 × 10^−3^	−0.488	−46.33	0.021	0.7977	79.77
	5.00 × 10−5	2.130	49.97	844	0.786	356.5	3.45 × 10^−3^	−0.563	−50.81	0.018	0.8462	84.62
	1.00 × 10−4	1.931	66.14	606	0.801	272.3	4.17 × 10^−3^	−0.614	−54.48	0.018	0.8838	88.38
	5.00 × 10−4	1.859	120.60	529	0.832	302.9	4.48 × 10^−3^	−0.590	−60.99	0.037	0.9363	93.63
	1.00 × 10−3	2.206	225.90	202	0.820	102.6	5.07 × 10^−4^	−0.721	−62.86	0.023	0.9660	96.60
Cr(iii)-L	1.00 × 10−5	2.172	35.11	1214	0.764	458.3	3.35 × 10^−3^	−0.501	−45.80	0.016	0.7812	78.12
	5.00 × 10−5	2.649	47.64	1012	0.771	410.6	2.37 × 10^−3^	−0.507	−47.56	0.020	0.8387	83.87
	1.00 × 10−4	1.906	56.05	885	0.801	419.8	3.88 × 10^−3^	−0.576	−53.44	0.024	0.8629	86.29
	5.00 × 10−4	2.925	80.76	719	0.795	344.7	1.92 × 10^−3^	−0.463	−52.66	0.028	0.9049	90.49
	1.00 × 10−3	1.898	168.80	348	0.856	215.5	5.60 × 10^−3^	−0.718	−65.82	0.036	0.9545	95.45

a *R*_s_ = solution resistance, *R*_ct_ = charge transfer resistant, *Y*_0_, *n* = constant phase elements, *C*_dl_ = double layer capacitance, S = slopes of bodes lines at intermediate frequencies, α° = phase angle, *τ* = relaxation time, *θ* = surface coverage, *η*_z_ = inhibition efficiency.

The low values of *C*_dl_ in case of metal complexes compared to those of the HCl solution are due to lower dielectric constant at the steel/electrolyte interface, which allows greater adsorption of Co(ii)-L or Cr(iii)-L on the metallic surface. It is worthy to note that lower *C*_dl_ values reflect more thickness of the adsorbed protecting layer. Furthermore, the drop in *Y*_o_, as the complex concentrations increases, confirms the growth of the number of adsorbed molecules on the metallic surface. Low *C*_dl_ and high *R*_ct_ is usually associated with excellent corrosion resistance. Moreover, the efficiency was increased gradually as a function of the addition of the complex concentrations. The uppermost *R*_ct_ is 225.90 and 168.80 Ω cm^2^ for Co(ii)-L and Cr(iii)-L complexes, respectively returning a maximum efficiency of 96.60 and 95.45% at 1.00 × 10^−3^ M for both complexes.

Only one peak that can be seen in the Bode and phase angle graphs (Fig. 8b) suggesting the presence of one time constant, accordingly the corrosion process takes place in a single phase under charge transfer control. The relaxation time constant (*τ*) was estimated from the product of *R*_ct_ and *C*_dl_ as following:^54^

6 *τ* = *R*_ct_ × *C*_dl_

The obtained values of *τ* was enhanced by the addition of the complex molecules from 0.017 to 0.037 s which can be explained by the fact that accumulation of concentrated concentrations of Co(ii)-L and Cr(iii)-L requires more adsorption time causing a delay of adsorption process.^55^ In addition, while the impedance modulus (*Z*_mod_) building up at low frequency region, the phase angle rises up (towards more negative values) and widens by the addition of the complex molecules indicating the successful adsorption to the metallic surface.^56^ The reinforcement of *Z*_mod_ in the low frequency area is associated with higher inhibition ability and lower corrosion rate. Furthermore, the slopes of *Z* at the intermediate frequency region and consequently the phase angles are shifted towards −1 and −90, respectively by the addition of the complex molecules as an indication of building up capacitive performance.^57^

#### 3.2.5 Adsorption considerations

**Fig. 9:**
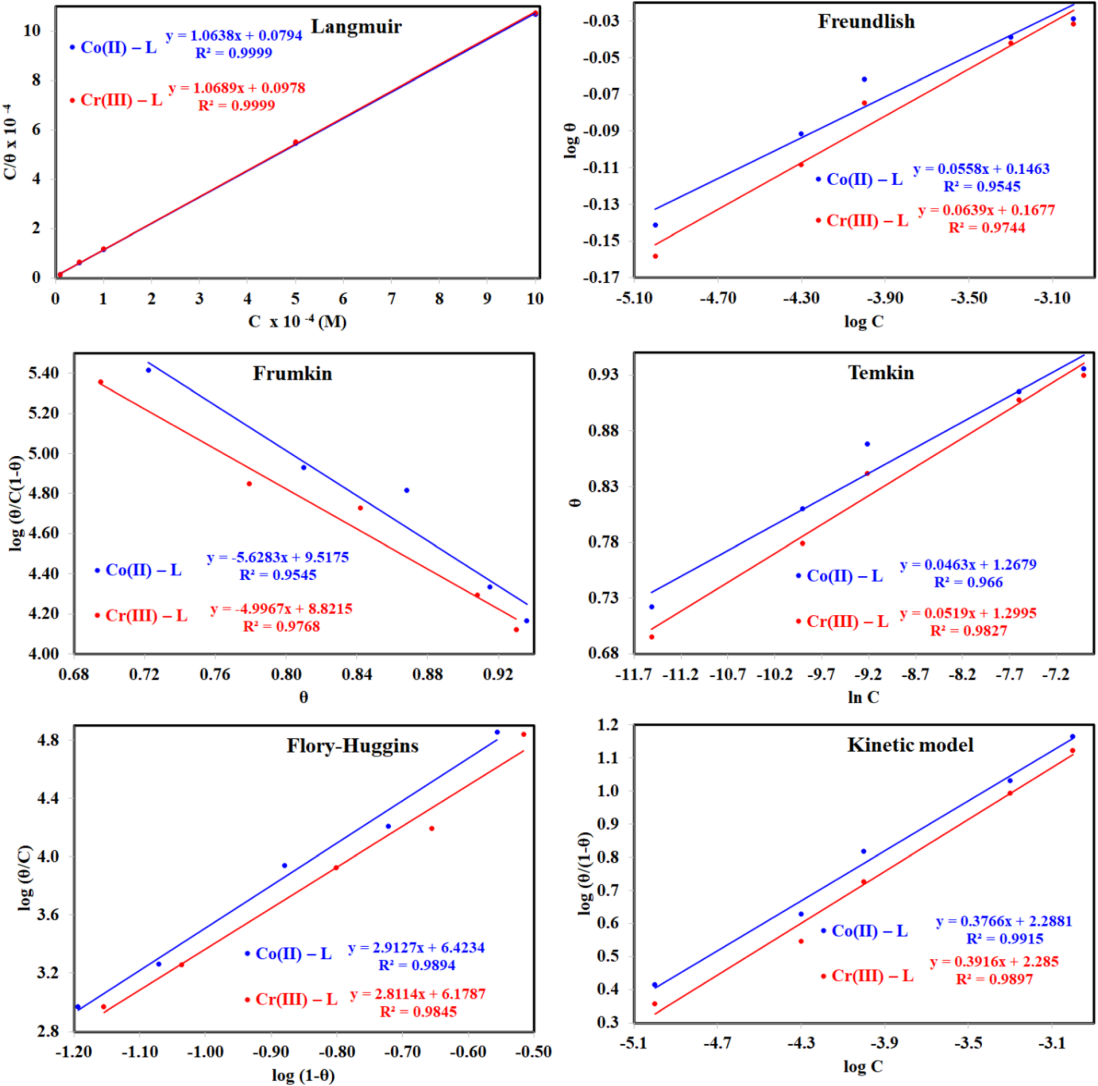
The different adsorption models for of Co(ii)-L and Cr(iii)-L compounds on the mild steel surface in 1.0 M HCl using data obtained from EFM measurements at 30 °C.

Different isotherms were used to fit the electrochemical data in order to get better understanding of the adsorption process of Co(ii)-L and Cr(iii)-L molecules on the steel surface. The isotherms formula and the corresponding extracted data such as slopes, intercepts, regression coefficient (*R*^2^), binding constant (*k*) and the adsorption free energy (Δ*G*) are presented in Table 6 while the corresponding plots are plotted in Fig. 9. In this study, we have fitted the electrochemical data to six adsorption models including kinetic-thermodynamic, Flory–Huggins, Temkin, Frumkin, Langmuir and Freundlich as they are most commonly used in the corrosion studies.^58^ In linear fitting, values of *R*^2^ are the key factor to determine the quality and accuracy of the fitting process.^59^*R*^2^ values in Table 6 reveal the following: Langmuir (*R*^2^ = 0.9999) > kinetic-thermodynamic (*R*^2^ = 0.99148) > Flory–Huggins (*R*^2^ = 0.9894) > Temkin (*R*^2^ = 0.98269) > Frumkin (*R*^2^ = 0.97678) > Freundlich (*R*^2^ = 0.97443). Therefore, the most accurate descriptions for the attachment of Co(ii)-L and Cr(iii)-L molecules on steel are both Langmuir and kinetic-thermodynamic (El-Awady) models.

**Table 6:** Adsorption isotherms models of the inhibitors with values of *R*^2^, slopes, intercepts, *K*_ads_, and Δ*G*_ads_ obtained by using data from electrochemical measurements^a^

Adsorption isotherm model	Linear form equation	Technique	Inhibitor	slope	intercept	*R* ^2^	*K*_ads_M^−1^	Δ*G*_ads_ kJ mol^−1^
Freundlich	log *θ* = log *K* + 1/*n* log C	EFM	Co(ii)-L	0.05579	0.14635	0.95451	1.4007	−10.97
			Cr(iii)-L	0.06392	0.16769	0.97443	1.4713	−11.09
Langmuir	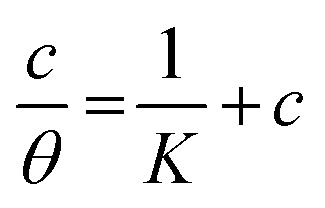	EFM	Co(ii)-L	1.06382	0.00001	0.99990	126 017	−39.70
			Cr(iii)-L	1.06890	0.00001	0.99988	102 277	−39.18
		PDP	Co(ii)-L	1.04375	0.00001	0.99993	189 929	−40.74
			Cr(iii)-L	1.06071	0.00001	0.99992	195 749	−40.81
		EIS	Co(ii)-L	1.03204	0.00001	0.99979	122 559	−39.63
			Cr(iii)-L	1.04690	0.00001	0.99934	98 695	−39.09
Frumkin	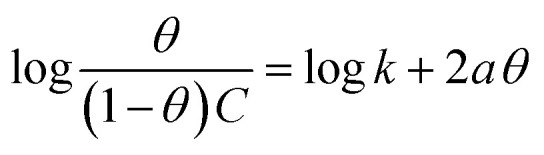	EFM	Co(ii)-L	−5.62834	9.51748	0.95454	3.2921 × 10^9^	−65.32
			Cr(iii)-L	−4.99669	8.82150	0.97678	6.6298 × 10^8^	−61.29
Temkin	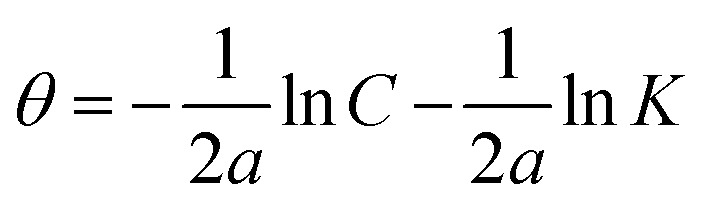	EFM	Co(ii)-L	20.87986	−26.77914	0.96605	0.2773	−6.89
			Cr(iii)-L	18.92702	−24.75165	0.98269	0.2704	−6.82
Flory-Huggins	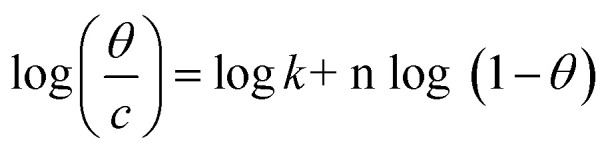	EFM	Co(ii)-L	2.91266	6.42345	0.98940	2.6512 × 10^6^	−47.38
			Cr(iii)-L	2.81143	6.17872	0.98449	1.5091 × 10^6^	−45.96
Kinetic-thermodynamic	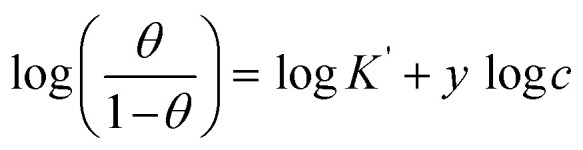	EFM	Co(ii)-L	0.37657	2.28815	0.99148	194.1548	−23.39
			Cr(iii)-L	0.39159	2.28504	0.98973	192.7719	−23.37

a *R* ^2^ = regression correlation coefficient, *K* = binding constant, *θ* = surface coverage, *c* = concentration.

The mathematical equation of Langmuir isotherm is:^60^7
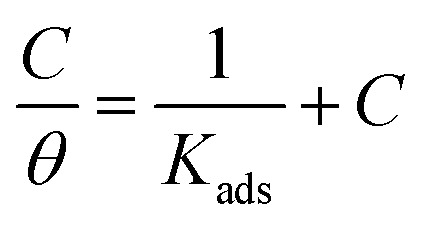


The obtained slopes for Co(ii)-L and Cr(iii)-L are almost unity (Table 6) which also confirms the Langmuir fit. This means that for Co(ii)-L and Cr(iii)-L, a mono molecular film was built up on the mild steel surface, and that the adsorption wasn't affected by the spatial interaction of nearby adsorbed species on the surface.^61^ According to Langmuir model, the number of adsorption locations on the surface are constant, each location holds only one adsorbed species, there is no interaction between sites, and the adsorption energies of all locations are identical.^62^

The mathematical equation of kinetic-thermodynamic (El-Awady) models is:^63^8
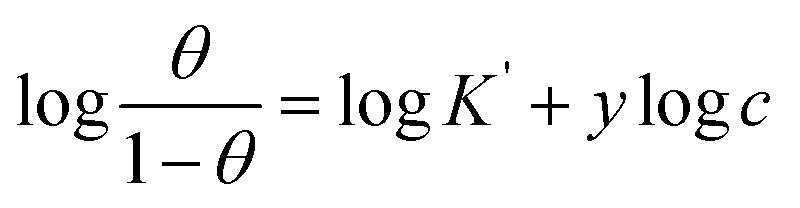


The reciprocal of *y* is a critical factor to determine the number of surface dangling water molecules that can be replaced by a single complex molecule. In the current study, 1/*y* is more than unity (2.66 for Co(ii)-L and 2.55 for Cr(iii)-L) which reveals that each complex molecule can be adsorbed by replacing more than one H_2_O molecule and therefore occupy more than one active site.^64^

Moreover, the spontaneity of adsorption and the nature of bonding with the metallic surface were addressed using Gibbs free energy, Δ*G*, according to the following equation:^65^

9 Δ*G*_ads_ = −*RT ln* 55.5 *K*_ads_

(*R* = 8.314 J mol^−1^ K^−1^, *T* = 303.15 K, [H_2_O] = 55.5 mol L^−1^)

A negative sign for Δ*G* was obtained from Langmuir fit for each of EFM, PDP and EIS data (Table 6) which confirms that the attachment of Co(ii)-L or Cr(iii)-L molecules on steel surface is the favorable mode. In addition, Table 6 shows that Δ*G* values for Co(ii)-L or Cr(iii)-L are ranged between −39.09 kJ mol^−1^ and −40.81 kJ mol^−1^, which confirms that the molecules undergo spontaneous mono layer chemisorption on the metallic surface.

### 3.3 Surface examination

#### 3.3.1 Scanning electron microscope (SEM)

**Fig. 10:**
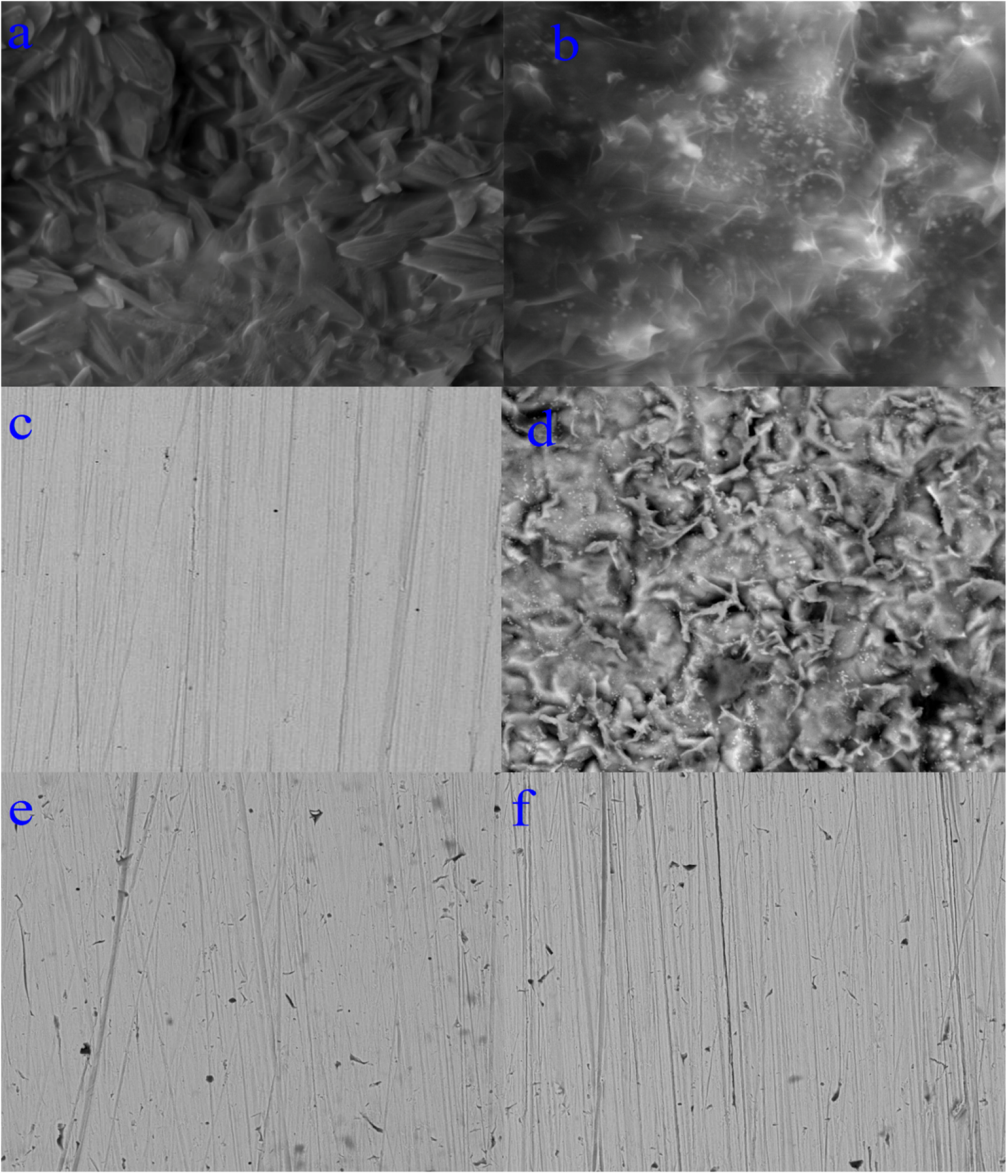
SEM images of (a) Co(ii)-L, (b) Cr(iii)-L, (c) polished steel, (d) (steel/1 M HCl), (e) (steel/1 M HCl/Co(ii)-L) and (f) (steel/1 M HCl/Cr(iii)-L).

The morphology of the synthesized complexes as well as steel in 1.0 M HCl (in absence and in presence of the complexes) was examined using an emission scanning electron microscope (SEM) as shown in Fig. 10(a–f). The SEM pictograph of Co(ii) complex (Fig. 10a) show a homogeneous matrix with an ideal shape of uniform phase material. The SEM examination revealed that crystals of Cr(iii) complex (Fig. 10b) were grew from a single molecule to many molecules in an aggregate distribution with particle sizes within few nanometers. The SEM picture in Fig. 10c reveals an abraded steel surface that seems to be smooth and clean, however in Fig. 10d, the steel surface was severely damaged by the immersion of steel in the corrosive solution. Nevertheless, in the presence of either Co(ii)-L or Cr(iii)-L complex (Fig. 10e and f), the steel surface was significantly less impacted and the damage is apparently lower, which suggests that steel samples containing Co(ii)-L or Cr(iii)-L molecules should have been covered with a layer that offers a magnificent level of protection against corrosive assault.^66^

#### 3.3.2 X-ray diffraction studies (XRD)

**Fig. 11:**
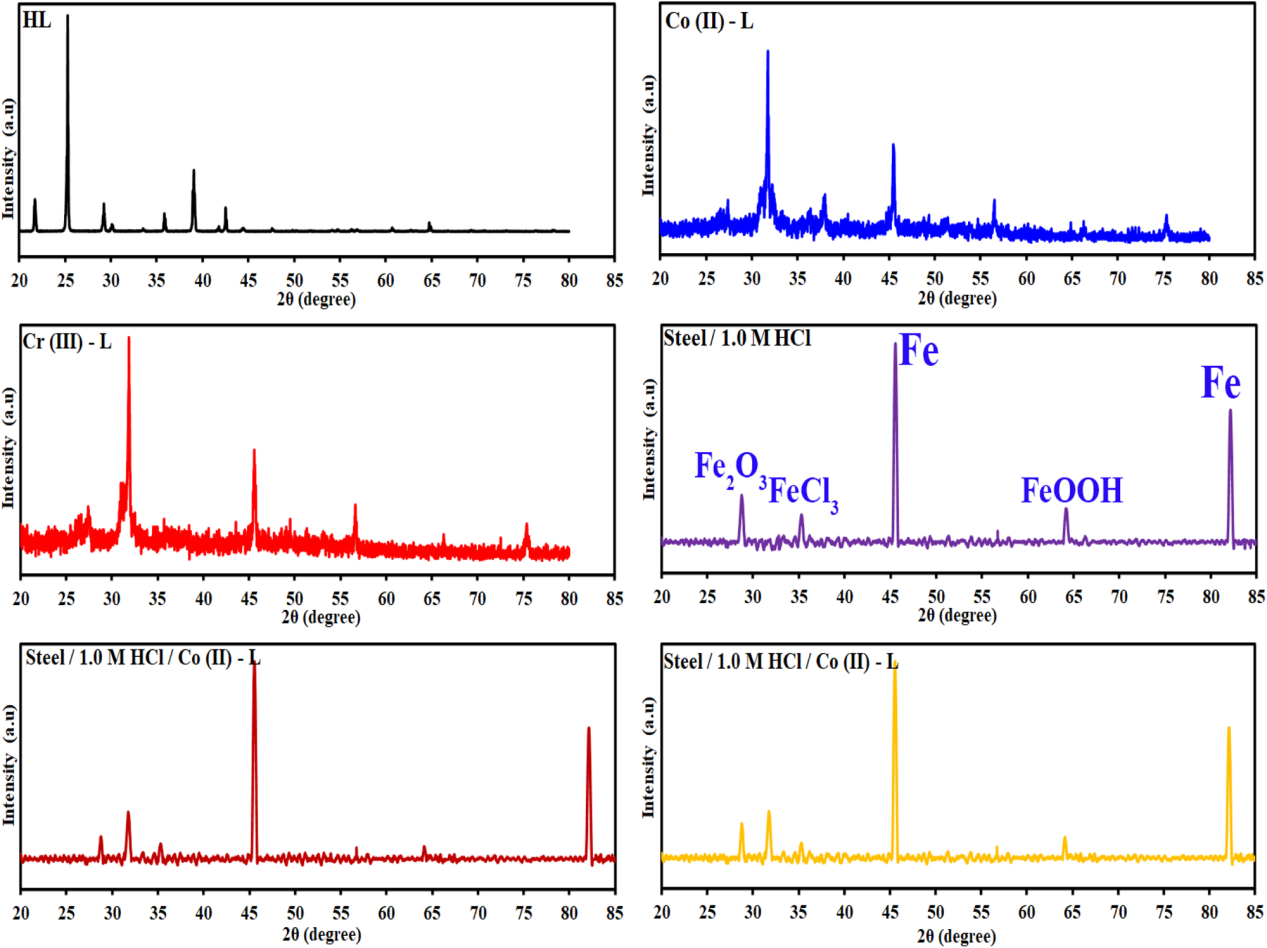
XRD patterns of the HL ligand, Co(ii)-L, Cr(iii)-L, (steel/1 M HCl), (steel/1 M HCl/Co(ii)-L) and (steel/1 M HCl/Cr(iii)-L).

The HL ligand, Cr(iii) and Co(ii) complexes were subjected to X-ray diffraction (as shown in Fig. 11) across a scanning range of 2*θ* = 10–80° to identify the kind of crystal system, lattice parameters, and information regarding unit cell structure and miller indices. In general, sharp, narrow, and strong peaks indicate high purity and crystallinity. The diffraction of HL exhibited peaks at 2*θ* [*d* value (Å)] = 21.96(4.09), 25.27(3.52), 35.79(2.51), 35.91(2.51), 38.89(2.31) 39.01(2.31), 42.49(2.13), 64.78(1.44) and 64.92(1.44)]. The diffraction of Co(ii) complex exhibited diffraction peaks at 2*θ* [*d* value (Å)] = 27.52(3.24), 31.82(2.81), 45.51(1.99), 56.64(1.41) and 75.37(1.26)]. The diffractogram of Cr(iii) complex indicated main peaks at 2*θ* [*d* value (Å)] = 19.0272(4.66), 30.96(2.88), 31.72(2.82), 32.37(2.766), 36.17(2.48), 45.48(1.99), 51.15(1.79), 52.86(1.73), 56, 45(1.63), 66.26(1.41), 71.91(1.31), 75(126)]. The average crystalline size (*t*) of the samples was estimated from XRD data using the simple Debye–Scherrer eqn (10):10
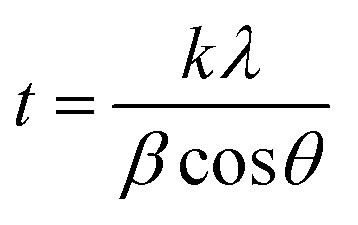
where, *t* is the crystallite size in (nm), *k* is a constant dependent on crystallite shape and equals to 0.89, *λ* is the wavelength of X-ray, *θ* is the diffraction angle in (degrees) and *β* is the full diffraction peak width at half maximum intensity (FWHM) in (radians). Table 7 displays the mean crystallite sizes which was calculated using the Scherrer equation. The values show that the sizes of the produced materials are within nano-scale dimensions. In addition, the findings in Table 7 show that the complexes have crystalline peaks.

**Table 7:** The average crystallite size of the synthesized complexes estimated from XRD pattern

Complexes	2*θ* (degree)	*d* value [Å]	Rel. Int. [%]	(FWHM)^a^	Average crystallite size (nm)	(*hkl*)
HL	25.2477	3.52751	100	0.0787	17.467	211
Cr(iii)-L	31.7176	2.82118	100	0.1181	7.251	111
Co(ii)-L	31.8197	2.81236	100	0.1968	12.081	210

a The maximum diffraction patterns according to the highest value of intensity.

Fig. 11 contains also the XRD patterns of the surface of the mild steel coupons immersed in the aggressive solutions in the absence and in presence of the inhibitors. For the steel/1.0 M HCl system, iron (Fe) and the iron corrosion products, like iron oxides (Fe_3_O_4_, FeOOH) and iron chlorides (FeCl_3_) phases, are clearly visible in the patterns obtained. Peaks at 2*θ* = 45.59°, 82.19° can be assigned to the metallic iron while the other corrosion products like oxides and chlorides was appeared at 2*θ* = 28.83°, 35.37° and 64.29°. In the presence of either Co(ii)-L or Cr(iii)-L molecules, we can notice that the intensity of the oxides and chlorides peaks have been diminished. This confirms that the designed complexes can protect the steel surface against corrosion. Also, the appearance of new peaks at 2*θ* = 31.87° and 31.97° related to Co(ii)-L and Cr(iii)-L molecules confirms the adsorption of these molecules at the interface between steel and the aggressive solution.

### 3.4 Molecular reactivity

#### 3.4.1 Global reactivity descriptors

The global reactivity descriptors answer the question; is the interaction between a particular ligand and a metal d-orbitals feasible? The answer is usually yes for common organic ligands as they usually possess a band gap with few electron volts and low ionization potentials due to the availability of the electrons, energetically up, in the π-system in addition to the common hetero atoms.^67^ The same concept applies for the interaction between the metal complex (as a corrosion inhibitor) and the mild steel. The availability of electrons for either metal–ligand or inhibitor-metal interactions depends on the eigenvalues of the highest occupied molecular orbital (HOMO) and lowest unoccupied molecular orbital (LUMO) and the reactivity descriptors that could be derived from these values such as ionization potential (IP), electron-affinity (EA), chemical hardness, *etc.*^68^ Therefore, it is important to accurately calculate the eigenvalues of HOMO and LUMO which is somehow tricky.

In a study on different types of organic and inorganic molecules, Zhang and Musgrave *et al.*^69^ reported that DFT calculations underestimates the eigenvalue of HOMO by average absolute error of 3.10 eV while it fails to predict LUMO eigenvalue. They have suggested that the eigenvalue of HOMO can be corrected using the formula: –HOMO_corr_ = *A* + *B*(−HOMO_cal_) where *A* = 1.42 and *B* = 1.20 being the correlation parameters of B3LYP functional.^69^ In addition, the eigenvalue of LUMO could be corrected using the formula: LUMO = HOMO + Δ*E*_gap_ where Δ*E*_gap_ could be obtained accurately from time dependent (TD) calculations. Table 8 lists the calculated band gap, eigenvalue of both HOMO and LUMO and the corresponding reactivity descriptors which was calculated as suggested by (ref. ^69^). As expected for a moderate size organic molecule, HL ligand show narrow band gap of 5.681 eV. Narrow gap causes wavefunctions of both HOMO and LUMO to be softly mixed and hence the molecule is easily polarized so that low amount of energy is sufficient to induce reallocation of ligand electrons into metal d-orbitals which results in a strong metal–ligand interaction and hence a stable complex, that can be a good candidate for suppressing corrosion of mild steel.^70,71^ The calculated band gap for Co(ii)-L and Cr(iii) complexes are 1.824 and 2.969 eV, respectively (Table 8) in good agreement with the calculated band gap of Co, Zn, Cd and Ni complexes which are 1.109, 1.458, 1.535 and 2.352 eV, respectively.^72^ The narrow band gap of Co(ii)-L and Cr(iii)-L complexes reflects high reactivity and therefore a strong interaction with mild steal surface and good adhering capabilities.

**Table 8:** Calculated electronic reactivity descriptors for HL ligand, Co(ii)-L and Cr(iii)-L metal complexes

Molecular Parameters^a^	Mathematical formula	HL ligand		Co(ii)-L		Cr(iii)-L	
		B3LYP	TD-B3LYP^b^	B3LYP	TD-B3LYP^b^	B3LYP	TD-B3LYP^b^
*E*_LUMO_	DFT calc	−1.37	−3.68	−2.26	−3.84	−6.20	−8.567
*E*_HOMO_	DFT calc	−6.62	−9.361	−3.54	−5.67	−8.43	−11.54
Δ*E*	(*E*_LUMO_–*E*_HOMO_)	5.245	5.681(5.012^c^)	1.28	1.824(0.8642^c^)	2.227	2.969(2.096^c^)
Ionization potential (IP)	−*E*_HOMO_	6.62	9.361	3.54	5.67	8.43	11.54
Electron affinity (EA)	−*E*_LUMO_	1.37	3.68	2.26	3.84	6.20	8.567
Electronegativity (*χ*)	(IP + EA)/2	3.995	6.520	2.9	4.76	7.32	10.05
Chemical potential (*μ*)	−*χ*	−3.995	−6.520	−2.9	−4.76	−7.32	−10.05
Chemical hardness (*η*)	(IP − EA)/2	2.625	2.840	0.64	0.915	1.115	1.49
Chemical softness (*σ*)	1/*η*	0.381	0.352	1.56	1.093	0.897	0.671
Global electrophilicity index (*ω*)	*χ* ^2^/2*η*	3.04	7.48	6.57	12.38	24.03	33.89

a All parameters in eV unit.

b All values are corrected according to the procedure in (ref. 62).

c The lowest excitation energy before correction.

The ionization potential (IP) – the negative value of HOMO – indicates how easily the electrons at HOMO can contribute to a chemical interaction.^71^ Low ionization potential enhances electron donation from the HOMO of the ligand to the virtual d-orbitals of the transition metal and therefore boosts up the coordination binding. A low IP of 9.361 eV for HL ligand (Table 8) reveal a good tendency of HL ligand to donate its HOMO electrons to the transition metal virtual orbitals. In addition, HL ligand show low chemical hardness 2.840 eV which predicts an efficient metal–ligand interaction. The low hardness is due to low band gap where in molecules with filled HOMO, the electronegativity separates the band gap into two parts which by combination gives the chemical hardness. Upon complex formation, the Co(ii)-L and Cr(iii)-L complexes show IP of 5.67 and 11.54 eV, respectively revealing better electron donating tendency of Co(ii)-L complex to the mild steal virtual orbitals than Cr(iii)-L in agreement with the electrochemical findings herein where Co(ii)-L complex has better electrochemical inhibition efficiency. The calculated chemical softness of HL ligand, Co(ii)-L and Cr(iii)-L are 0.352, 1.093 and 0.671, respectively (Table 8) showing that chelating with Cobalt enhances the reactivity of the ligand for better interaction with metals.

#### 3.4.2 Molecular orbital analysis

Molecular orbitals can be investigated either through frontier molecular orbital analysis (FMO) or natural bond orbital analysis (NBO). Orbital densities from FMO analysis are delocalized over many atoms, in other words, it represents the contribution of different atoms in a particular molecular orbital. For example, if we are interested in HOMO, we can determine which atoms constitute (by their densities) the HOMO. By this definition, it is useful to use it to validate chemical interactions and attribute specific atoms to these interactions. On the other hand, orbital densities from NBO analysis are localized or few-atom-centered. They are not treated like classical hybrid orbitals used in frontier molecular orbital analysis, but they are Lewis-like or hybrid-like orbitals. By this definition, they can be used to assign which atom has the priority to interact or in other words which is easier to donate electrons. FMO and NBO analysis are complementary molecular orbital investigations for metal–ligand interactions and therefore a vital tool in complex formation and corrosion studies.

**Fig. 12:**
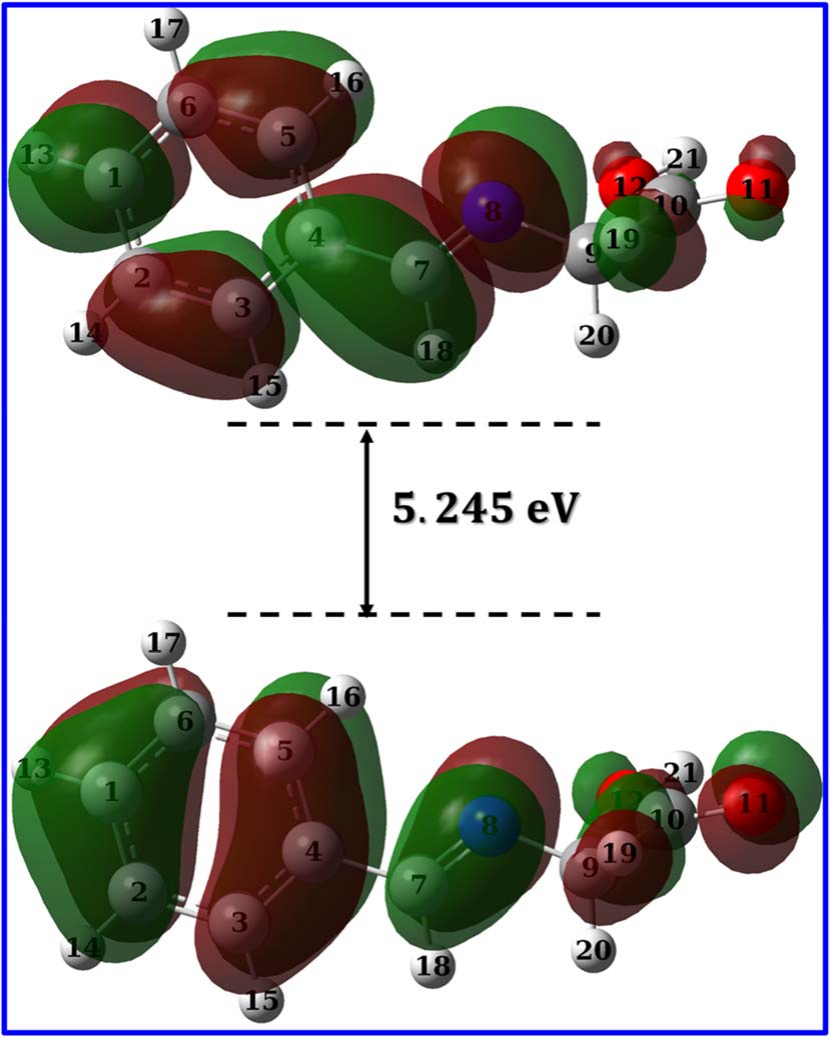
LUMO and HOMO densities of HL ligand at B3LYP/6-31g(d, p).

**Fig. 13:**
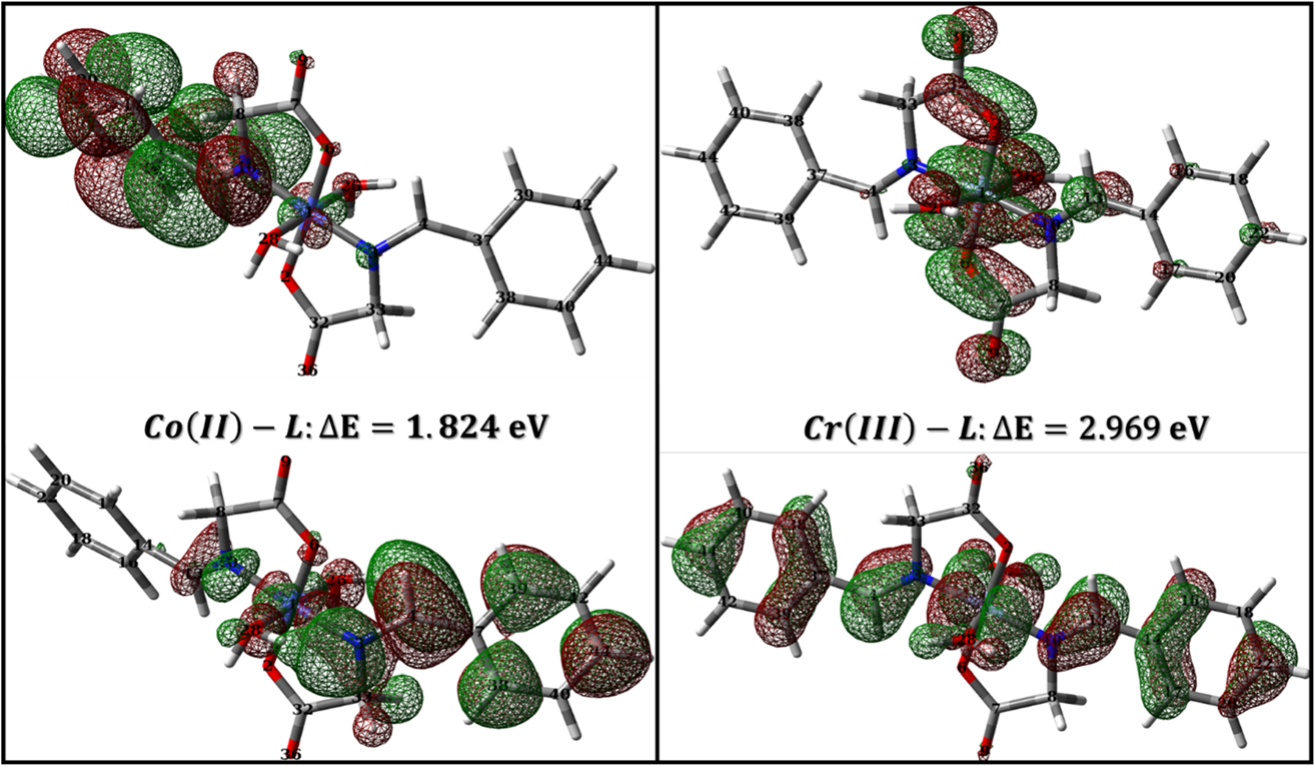
LUMO and HOMO densities of Co(ii)-L and Cr(iii)-L complexes.

Fig. 12 and 13 presents the FMOs (specifically HOMO and LUMO surface densities) for HL ligand and the corresponding metal complexes, respectively. It shows that the HOMO of HL ligand is delocalized mainly over the phenyl ring, the C_7_=N_8_ bond with less extent to O_11_ and C_9_ atoms. Furthermore, a negligible contribution of O_12_–H_21_ bond to HOMO can be noticed. N_8_ atom is in conjugation with the phenyl group; an electron rich group, with electron density in HOMO, would promotes the donation ability of N_8_ so that it easily captures a metal through a coordination bond. In addition, the lack of electron density contribution to HOMO from O_12_–H_21_ bond proves the acidity of H_21_ atom and supports the formation of the covalent bond between O_12_ and the metal. Overall, FMOs analysis ascribe the priority of electron donation to the phenyl ring and to the lone pair of N_8_ atom. While the later will be involved in a coordination bond with the transition metals (*i.e.*: ligand–metal interaction), delocalized electron density of phenyl group would provide a good donating center when applying the formed complex to the mild steel (*i.e.*: inhibitor–metal interaction). This is clearly appear in the FMOs of the Co(ii)-L and Cr(iii)-L complexes (Fig. 13) where the HOMO is delocalized over the phenyl group of the ligand molecule that would forms an effective adhering blocking film to the corrosion of the mild steel surface.

**Fig. 14:**
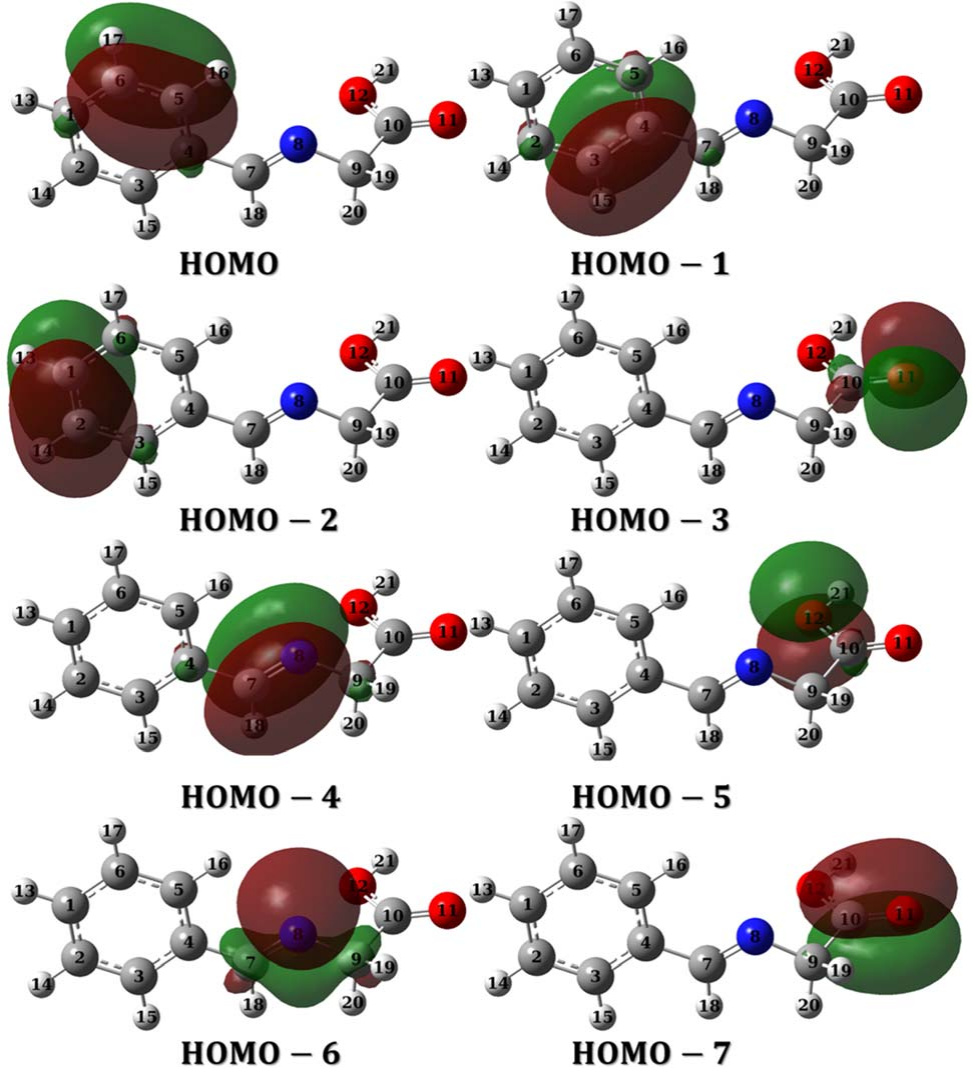
NBOs densities of HL ligand from HOMO to HOMO-7.

To evaluate the priority of individual atoms/groups, to share their electrons with metal d-orbitals, the NBOs were analyzed. Table S1† lists the orbital hybridization scheme for suggested points of interactions ordered by the priority of electron donating ability along with their corresponding eigenvalues in eV. The corresponding surface densities are provided in Fig. 14 with the same order. According to Table S1,† hybridizations of the HL ligand are in the following order: BD(2)C_5_–C_6_ > BD(2)C_3_–C_4_ > BD(2)C_1_–C_2_ > LP(2)(O_11_) > BD(2)C_7_–C_8_ > LP(2)(O_12_) > LP(1)(N_8_) > BD(2)C_10_–C_11_. These hybridizations correspond to the orbitals from HOMO to HOMO-7 (Fig. 14). This order corresponds to their ability to reallocate their electron density into d-orbitals of the metal; for example: the electrons of the π bond of the binding orbital BD(2)C_5_–C_6_ lies on the top of the natural bond orbitals, which is the HOMO, and hence easiest donation can take place from it, followed by the other two binding orbitals of the phenyl π-system. Nevertheless, on NBOs energy basis, the lone pair of O_12_ atom has priority to donate electrons to the metal slightly more than the lone pair of N_8_ atom (by ≈ 0.555 eV), however, two other factors favor that N_8_ would form the coordination bond rather than O_12_: (i) steric hindrance due to the covalent bond with O_11_, and (ii) hybridization where the lone pair of oxygen is pure p orbital (99.88%), the lone pair of nitrogen is sp^2^ (ref. 30) having 30.30 and 69.57% s and p-character, respectively. Increasing s-character in the lone pair of nitrogen atom would increase the angle to the adjacent bond and therefore enhances the formation of a stable five membered ring. Furthermore, Table 9 and 10 list the NBOs of the Co(ii)-L and Cr(iii)-L complexes, respectively with their corresponding orbital densities presented in Tables S2 and S3,† respectively. The NBOs of Co(ii)-L show twenty two positions that can share their electrons with mild steel surface against twenty positions for Cr(iii)-L (two of them arises from water molecules). This result should favor the Co(ii)-L complex more slightly than Cr(iii)-L complex as a corrosion inhibitor which agree with the electrochemical results. As expected, lone pair of nitrogen atoms comes late in NBOs due to coordination.

**Table 9:** Calculated NBOs of Co(ii)-L at expected inhibitor–metal interactions

Type^a^	Occupancy	Energy	NBO	s% Atom 1	p% atom 1	d% atom 1	p% atom 2
LP(1)C_37_	0.54197	−0.09781	p^1.00^	0.00	99.99	—	—
LP(1)C_44_	0.58409	−0.10833	p^1.00^	0.00	99.99	—	—
LP(2)O_9_	0.91997	−0.21562	p^1.00^	0.00	99.70	—	—
LP(2)O_36_	0.92121	−0.22713	p^1.00^	0.00	99.72	—	—
BD(2)C_38_–C_40_	0.83318	−0.23161	0.6667 p + 0.7454 p	—	99.95	—	99.97
BD(2)C_39_–C_42_	0.83337	−0.23199	0.6652 p + 0.7467 p	—	99.95	—	99.97
BD(2)C_4_–C_37_	0.88666	−0.25085	0.8260 p + 0.5637 p	—	99.86	—	99.58
LP(2)O_6_	0.86094	−0.26609	p^1.00^	—	99.86	—	—
BD(2)C_18_–C_22_	0.81547	−0.26962	0.7148 p + 0.6993 p	—	99.96	—	—
BD(2)C_14_–C_16_	0.80988	−0.27055	0.7284 p + 0.6851 p	—	99.97	—	99.96
BD(2)C_17_–C_20_	0.82987	−0.27265	0.7026 p + 0.7116 p	—	99.96	—	99.96
LP(3)Co	0.96777	−0.27740	d^1.00^	—	—	99.98	—
LP(2)Co	0.98324	−0.27778	d^1.00^	—	—	99.96	—
LP(1)Co	0.98984	−0.28621	d^1.00^	—	—	99.99	—
LP(2)O_2_	0.86956	−0.29382	p^1.00^	—	98.82	—	—
BD(2)N_3_–C_4_	0.94508	−0.30489	0.8425 p + 0.5387 p	—	99.88	—	99.67
LP(1)N_3_	0.91346	−0.33453	sp^3^ (ref. 68)	21.37	78.61	—	—
BD(2)N_10_–C_13_	0.97499	−0.36680	0.7939 p + 0.6081 p	—	99.83	—	99.72
BD(2)C_7_–O_9_	0.99703	−0.36899	0.5475 p + 0.8368 p	—	98.41	—	97.92
LP(1)N_3_	0.81589	−0.38646	sp^1^ 94	33.97	65.99	—	—
BD(1)Co–N_3_	0.96925	−0.40325	0.5732 sp^1^ (ref. 36) d^22^ + 0.8194 sp^8^	4.18	5.69	90.11	—
LP(1)N_10_	0.83141	−0.41440	sp^2^ (ref. 61)	27.72	72.28	—	—

a LP(1): refers to first lone pair, LP(2): second lone pair, *etc.* BD(1): bonding orbital of a single bond, BD(2): for double bond.

**Table 10:** Calculated NBOs of Cr(iii)-L at expected inhibitor–metal interactions

Type^a^	Occupancy	Energy	NBO	s% Atom 1	p% Atom 1	d% Atom 1	p% Atom 2
LP(1)C_40_	0.48963	−0.20986	p^1.00^	0.00	99.97	—	—
LP(1)Cr	0.85752	−0.30772	d^1.00^	—	—	100	—
LP(2)O_9_	0.91521	−0.34278	p^1.00^	0.00	99.71	—	—
LP(2)O_36_	0.91472	−0.34363	p^1.00^	0.00	99.70	—	—
BD(2)C_18_–C_22_	0.80123	−0.34840	0.7226 p + 0.6913 p	—	99.96	—	99.95
BD(2)C_40_–C_44_	0.80094	−0.34860	0.7182 p + 0.6959 p	—	99.96	—	99.95
BD(2)C_14_–C_16_	0.80248	−0.35711	0.7362 p + 0.6767 p	—	99.98	—	99.96
BD(2)C_17_–C_20_	0.83148	−0.35724	0.7041 p + 0.7101 p	—	99.96	—	99.96
BD(2)C_37_–C_38_	0.80671	−0.35921	0.7353 p + 0.6778 p	—	99.99	—	99.96
LP(1)Cr	0.98744	−0.39234	d^1.00^	—	—	99.98	—
LP(2)O_2_	0.85209	−0.40968	p^1.00^	—	99.06	—	—
LP(2)O_6_	0.85199	−0.41170	p^1.00^	—	98.70	—	—
BD(2)C_7_–O_9_	0.99247	−0.47161	0.5675 p + 0.8233 p	—	99.83	—	99.65
BD(2)C_32_–O_36_	0.99282	−0.47311	0.5684 p + 0.8227 p	—	99.82	—	99.63
BD(2)N_10_–C_13_	0.97273	−0.47507	0.8140 p + 0.5808 p	—	0.97273	—	99.82
BD(2)N_3_–C_4_	0.97326	−0.47565	0.8153 p + 0.5791 p	—	99.93	—	99.82
LP(1)O_28_	0.97593	−0.51948	p^1.00^	—	99.64	—	—
LP(1)O_26_	0.97121	−0.52641	p^1.00^	—	99.62	—	—
LP(1)N_3_	0.82365	−0.54312	sp^2^ (ref. 43)	29.12	70.88	—	—
LP(1)N_10_	0.82561	−0.54313	sp^2^ (ref. 41)	29.34	70.66	—	—

a LP(1): refers to first lone pair, LP(2): second lone pair, *etc.* BD(1): bonding orbital of a single bond, BD(2): for double bond.

#### 3.4.3 Local reactivity descriptors

Molecular regions having tendency for an electrophilic or nucleophilic attack could be configured by the aid of molecular electrostatic potential (MEP) map. Using B3LYP/6-31G(d,p) calculations and GaussView 6 graphical interface, MEP map were extracted for HL ligand, Fig. S3.† In MEP map, regions with low electrostatic potential energy (*i.e.*: electron-rich) is indicated by red color while those with high electrostatic potential energy (*i.e.*: electron-poor) is indicated by blue color. The presented map in Fig. S3† show that HL ligand has one extreme electron-rich region (red region) around O_11_ atom, one extreme electron-poor region (blue region) around H_21_ atom, moderate electron content region (fade red color) around N_8_ atom and uniform electron content region (green color) around the phenyl group. It is worth to note that the magnitude of electrophile and nucleophile sites doesn't necessarily reflects the net reactivity of these sites. The other factors like energy of the site and steric hindrance are important factors which couldn't be inferred from the MEP map. Nevertheless, it still very informative regarding regions where the electrophile (*i.e.* the transition metal) may contact the ligand. In HL ligand, the electron-rich sites are those surrounding carboxylic oxygen while electron-poor sites are those surrounding carboxylic hydrogen, indicating a notable acidity of H_21_ atom, which is important for complex formation through a covalent bond with O_12_ atom. On the other hand, the green colored sites of the π-system of phenyl rings indicates uniform distribution of electronic density content.

Furthermore, Fukui functions have been used widely for the estimation of local reactivity within a molecule by determining the ability of an atom to either capture an electronic density of an nucleophile or release off part of its electronic density to an electrophile species.^73^ On this basis, atoms can be classified as either local electrophile or nucleophile site and hence Fukui functions can be very informative regarding ligand–metal interactions. The analysis probes the energy changes when molecules approach each other, therefore takes into accounts mutual interactions between the approaching reactants. To do so, the second derivative of energy is taken with respect to; (i) number of electrons around an atom, (ii) the external potential due to mutual interactions between molecules. This is defined mathematically as:

11 *f*(*r*) = ∂^2^*E*/∂*N* ∂*ν*

Although different approximations were used to calculate these derivatives using charge densities, definition of reactivity on an atom-by-atom basis using a “condensed” Fukui function is much convenient for such systems. So, the functions for electrophilic and nucleophilic attack are defined as the following:^74^

12 *f*^−^_*k*_ = [*q*_k_(N)−*q*_*k*_(N−1)]

13 *f*^+^_*k*_ = [*q*_k_(N + 1)−*q*_k_(N)]

where *q*_*k*_ is the charge on particular atom at atomic center *k*. Here, we adopted the Hirshfeld analysis to obtain atomic charges since it is corrected to bond orders and therefore much suitable for systems with multiple single-double conjugations. The calculated Fukui indices for the HL ligand molecule are listed in Table S4.† The electrophilic Fukui indices *f*^−^show that when metal approaches the HL ligand, N_8_ atom (*f*^−^ = 0.114) can have the largest electronic density contribution with the d-orbitals of the metal which is good evidence for the feasibility of formation of coordination bond through it. In addition, O_12_ atom has the lowest electrophilic indices (*f*^−^ = 0.007) while having nucleophilic indices (*f*^−^ = 0.031) indicating a nucleophilic site which can lose its acidic hydrogen and contact metal through a full negative charge. On the other hand, the nucleophilic and electrophilic Fukui indices for the O_11_ are 0.101 and 0.050, respectively which minimize its chances for contacting metal through a coordination bond.

## 4. Conclusions

The present work reported the synthesis of two mononuclear complexes by the chelation of Co(ii) and Cr(iii) ions with 2-(benzylideneamino)acetic acid (HL). Their structural details were presented and analyzed by several analytical and spectroscopic tools. The results showed that 2-(benzylideneamino) acetic acid functions as a bi-dentate ligand through azomethine nitrogen and carboxylate oxygen. The results of this investigation support that metal complexes have an octahedral shape. The prepared cobalt(ii) and chromium(iii) complexes were found to be an excellent inhibitor for steel corrosion. The adsorption of the two complexes on the metallic surface was found to be mixed type, controlled by the charge transfer mechanism and follows Langmuir isotherm model. The morphological surface analysis XRD and SEM proved the formation of a protective layer that retarded the corrosion reaction. The active regions for the metallic protection were confirmed from NBO analysis.

## Conflicts of interest

The authors declare that they have no known competing financial interests or personal relationships that could have appeared to influence the work reported in this paper.

## Supplementary Material

RA-012-D2RA06571A-s001
