# Supplementary material for: Effective corrosion inhibition of mild steel in hydrochloric acid by newly synthesized Schiff base nano Co(ii) and Cr(iii) complexes: spectral, thermal, electrochemical and DFT (FMO, NBO) studies
Source: RSC Adv. 2022 Nov 14;12(50):32488–507. doi: 10.1039/d2ra06571a (PMC9661184; doi:10.1039/d2ra06571a)
Supplement: RA-012-D2RA06571A-s001 [file RA-012-D2RA06571A-s001.pdf]

Electronic Supplementary Material (ESI) for RSC Advances.
This journal is © The Royal Society of Chemistry 2022

Supporting Information for

Effective Corrosion Inhibition of Mild Steel in Hydrochloric Acid by a Newly Synthesized Schiff Base Nano Co(II) and Cr(III) Complexes: Spectral, Thermal, Electrochemical and DFT (FMO, NBO) Studies

Saad Melhi[1], Mahmoud A. Bedair[2,3,*], Eid H. Alosaimi[1], Ayman A.O. younes[1], Walaa H. El-Shwiniy[1,4], Ahmed M. Abuelela[3]

[1]*Department of Chemistry, College of science, University of Bisha, P.O. Box 511, Bisha 61922, Saudi Arabia*
[2]*College of science and arts, University of Bisha, Al-Namas 61977, P.O. Box 101, Saudi Arabia*
[3]*Department of Chemistry, Faculty of Science (Men's Campus), Al-Azhar University, Nasr City 11884, Cairo, Egypt*
[4]*Department of Chemistry, Faculty of Science, Zagazig University, Zagazig 44519, Egypt.*

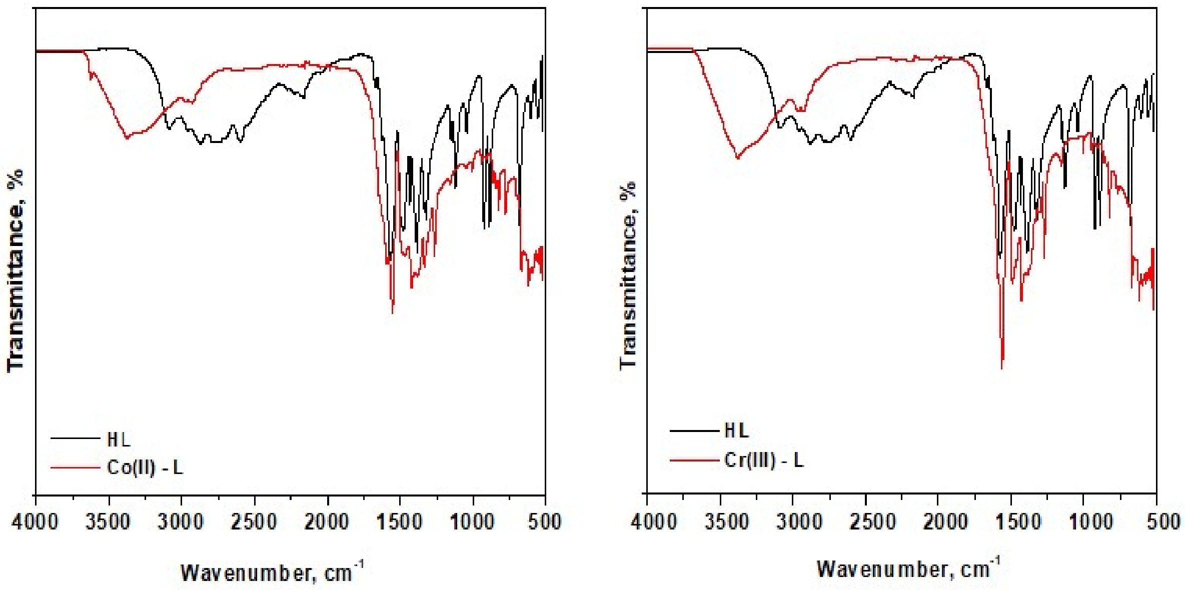

Fig. S1: Infrared spectra for **HL** ligand and its complexes.

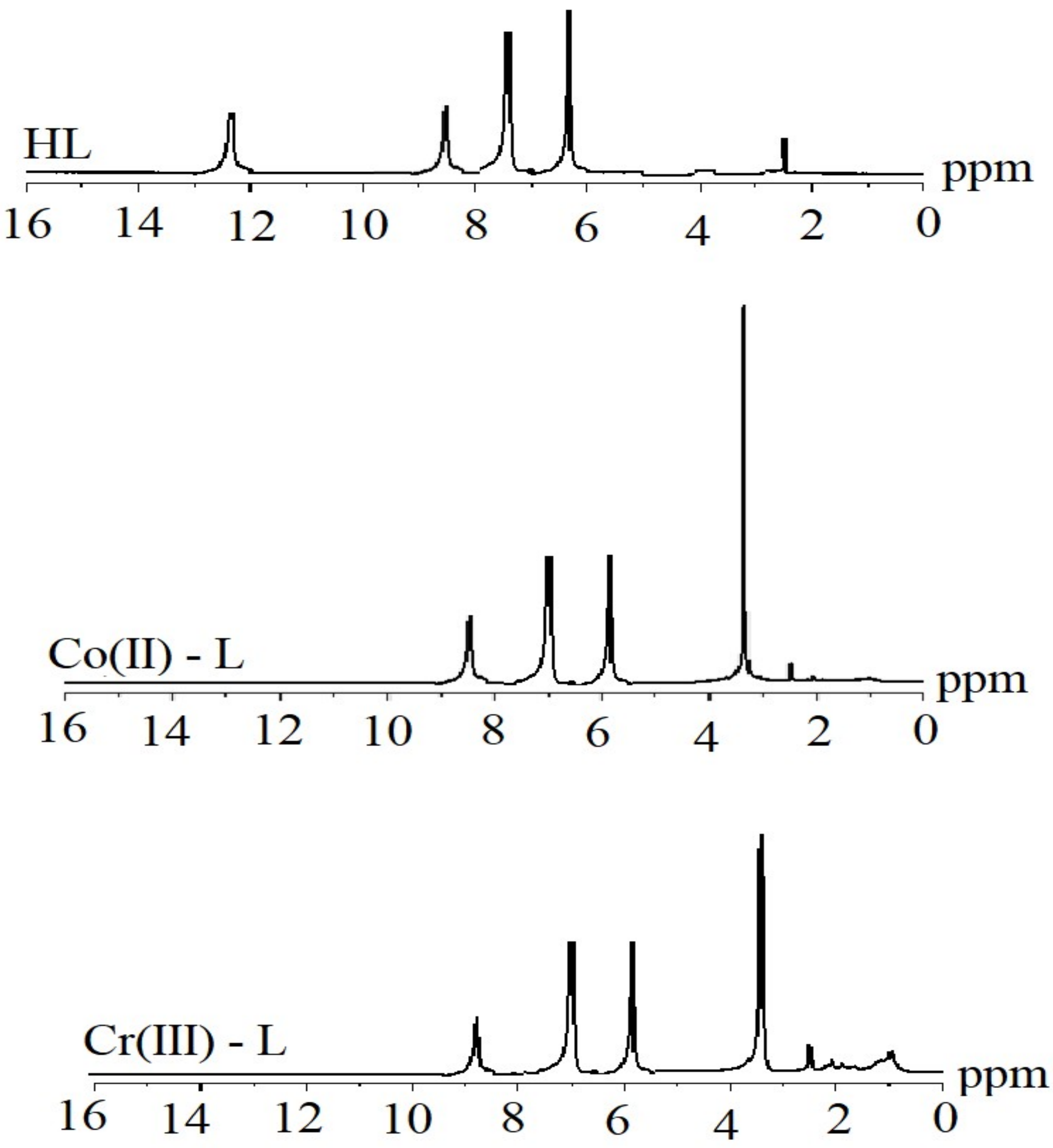

Fig. S2: ^{1}H NMR spectrum for HL and its complexes.

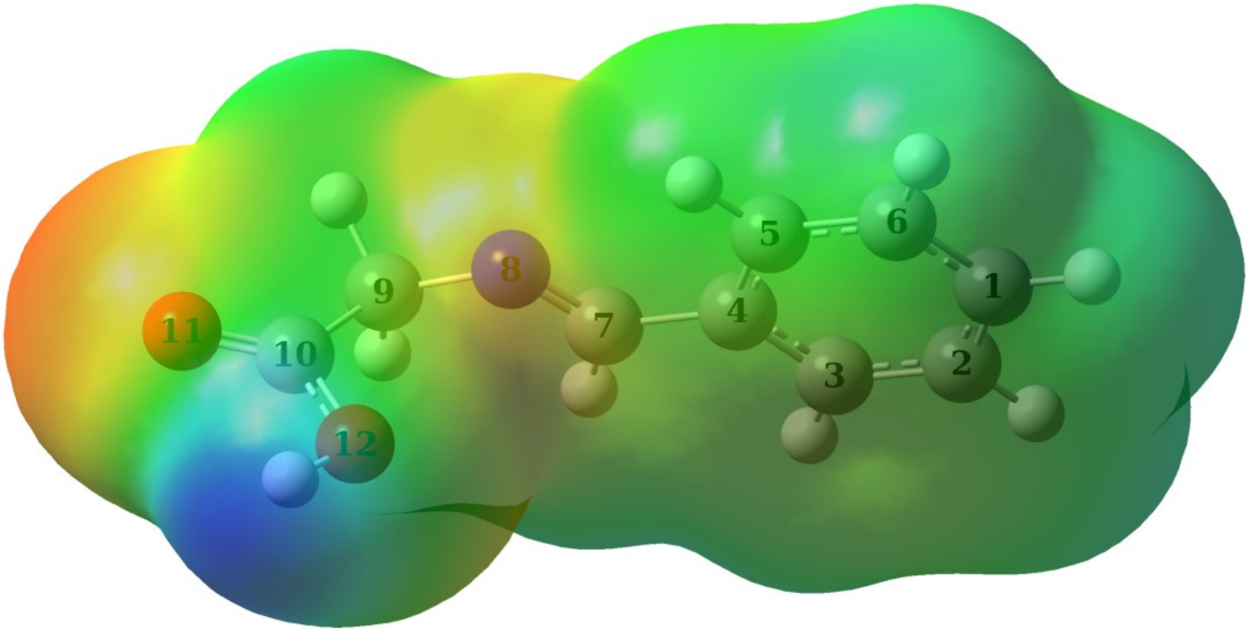

Fig. S3 Molecular electrostatic potential map of **HL** ligand.

Table S1 NBOs at inhibitor-metal interactions ordered according to their energies (highest to lowest).

Type*	Occupancy	Energy	NBO	s % (Atom 1)	p % (Atom 1)	s % (Atom 2)	p % (Atom 2)
BD(2)C_5-C_6	1.66370	-0.24638	0.6968 p + 0.7172 p	0.00	99.95	0.00	99.96
BD(2)C_3-C_4	1.63715	-0.24699	0.6961 p + 0.7180 p	0.00	99.96	0.00	99.98
BD(2)C_1-C_2	1.65809	-0.24871	0.7018 p + 0.7124 p	0.00	99.96	0.00	99.96
LP(2)(O_{11})	1.97575	-0.26031	$p^{1.00}$	0.01	99.73	-	-
BD(2)C_7-C_8	1.93923	-0.32113	0.6460 p + 0.7633 p	0.01	99.89	0.02	99.72
LP(2)(O_{12})	1.97654	-0.33105	$p^{1.00}$	00.00	99.88	-	-
LP(1)(N_8)	1.91109	-0.35146	$sp^{2.30}$	30.30	69.57	-	-
BD(2)C_{10}-C_{11}	1.99249	-0.39203	0.5543 p + 0.8323 p	0.40	99.40	0.60	99.05

*LP(1): refers to first lone pair, LP(2): second lone pair, etc. BD(1): bonding orbital of a single bond, BD(2): for double bond.

Table S2 Calculated NBOs densities of Co(II)-L at expected inhibitor-metal interactions.

**LP(1)C_{37}**	**LP(1)C_{44}**
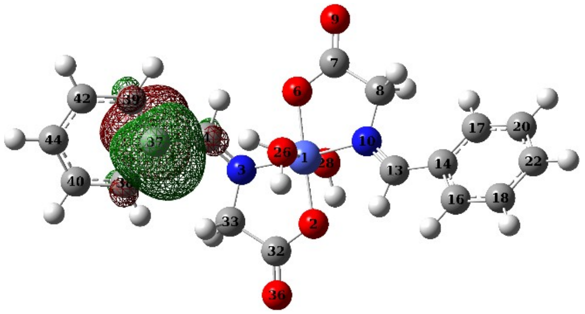	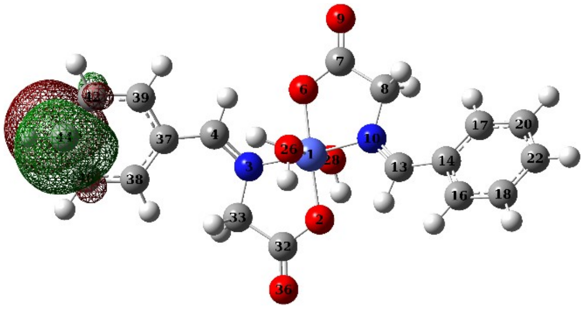
**LP(2)O_9**	**LP(2)O_{36}**
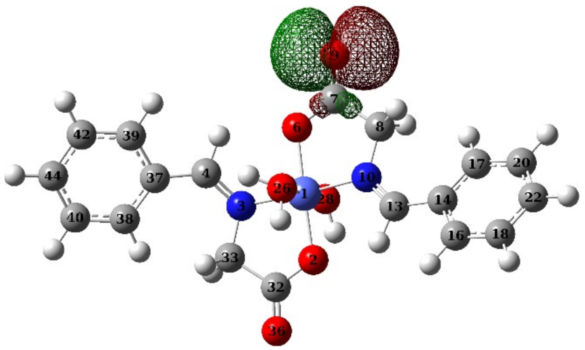	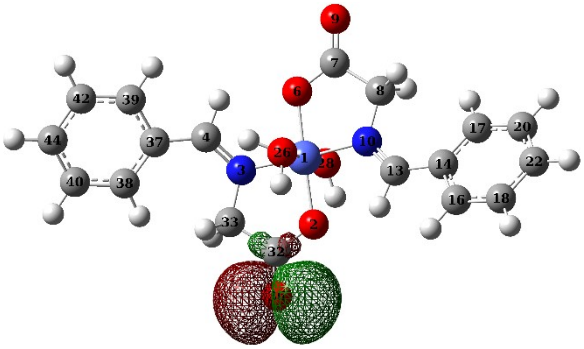
**BD(2)C_{38}-C_{40}**	**BD(2)C_{39}-C_{42}**
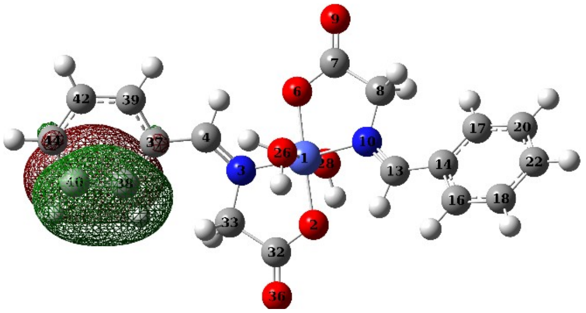	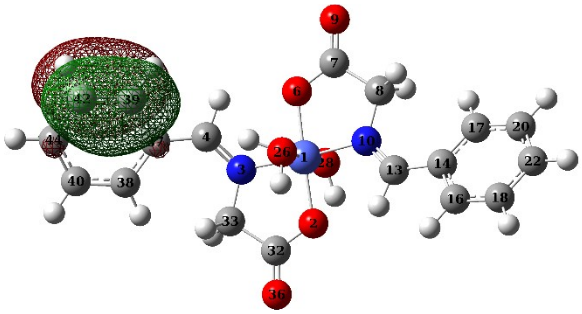
**BD(2)C_4-C_{37}**	**LP(2)O_6**
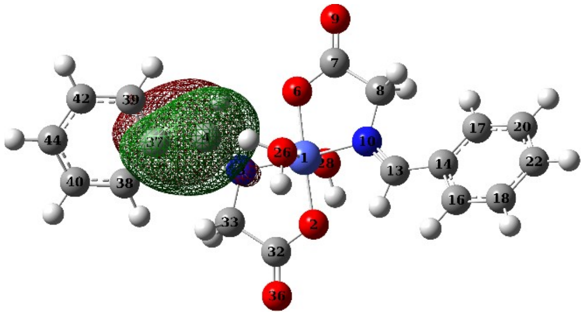	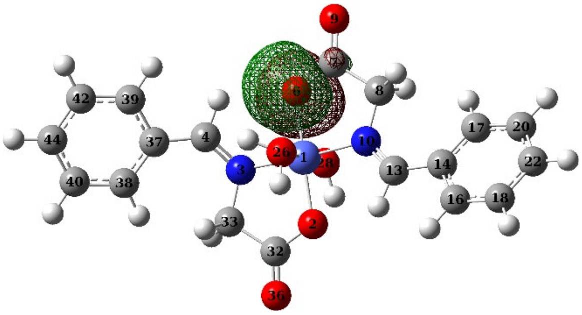
**BD(2)C_{18}-C_{22}**	**BD(2)C_{14}-C_{16}**
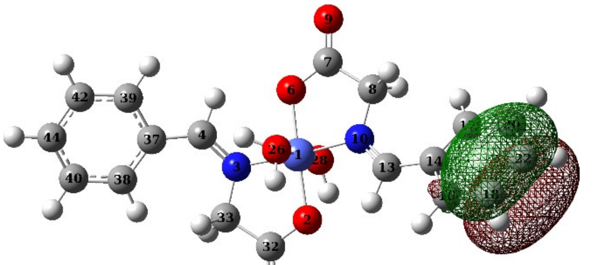	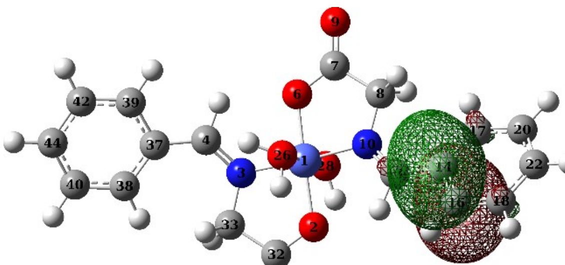

**BD(2)C_{17}-C_{20}**	**LP(3)Co**
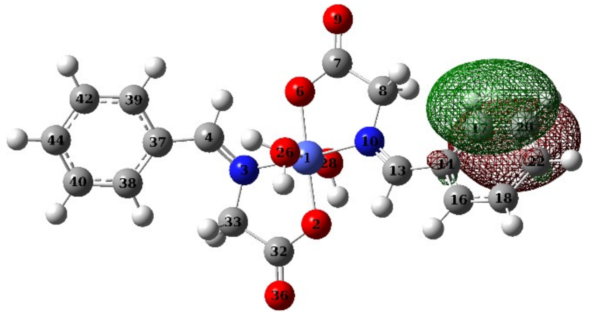	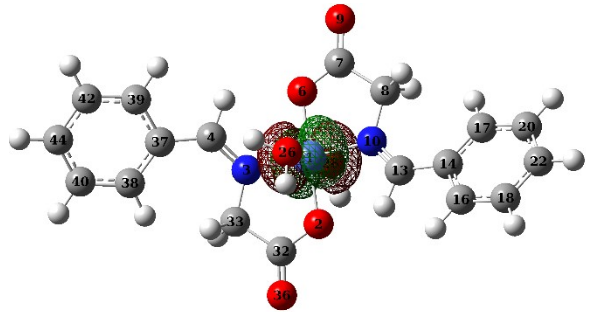
**LP(2)Co**	**LP(1)Co**
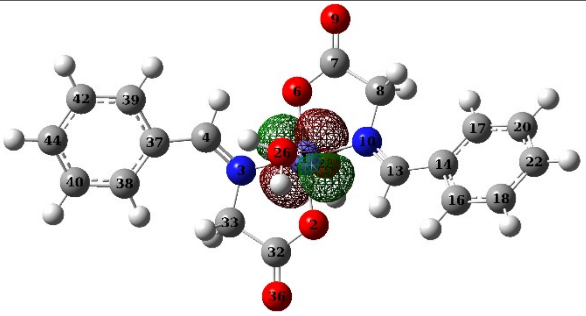	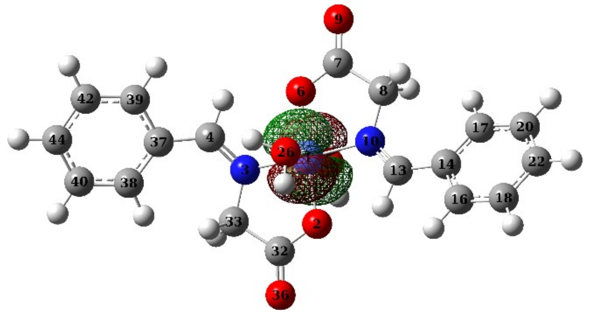
**LP(2)O_2**	**BD(2)N_3-C_4**
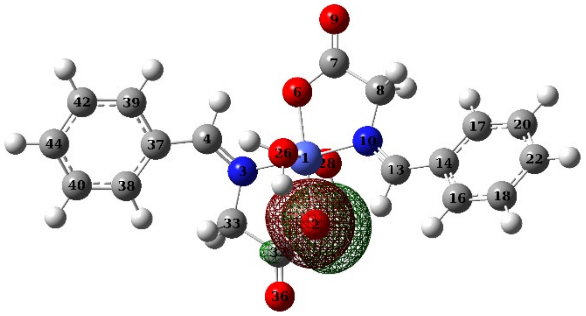	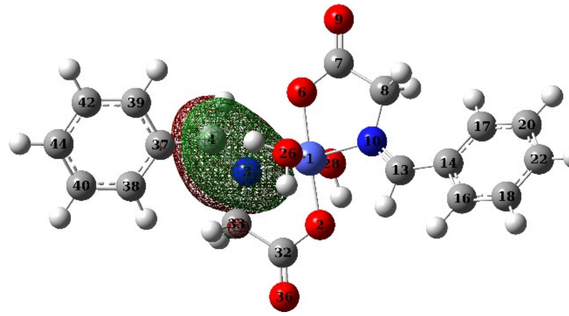
**LP(1)N_3**	**BD(2)N_{10}-C_{13}**
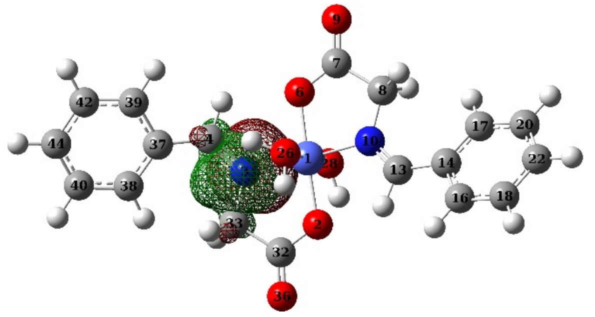	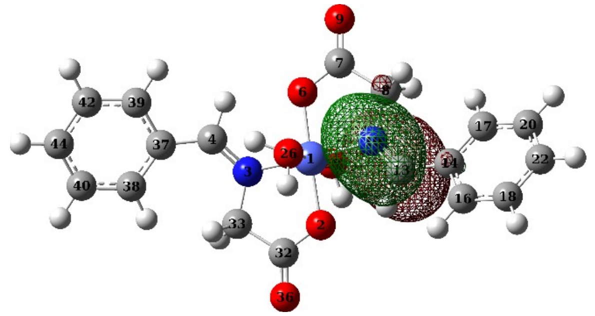
**BD(2)C_7-O_9**	**LP(1)N_3**

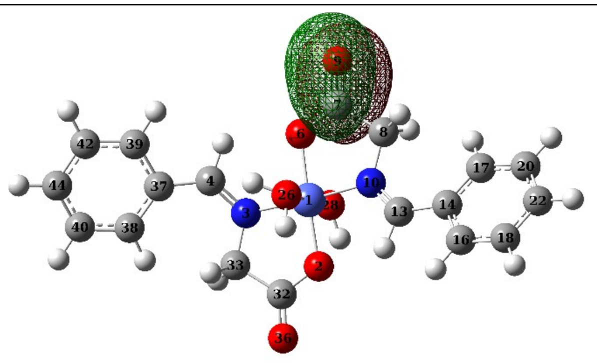

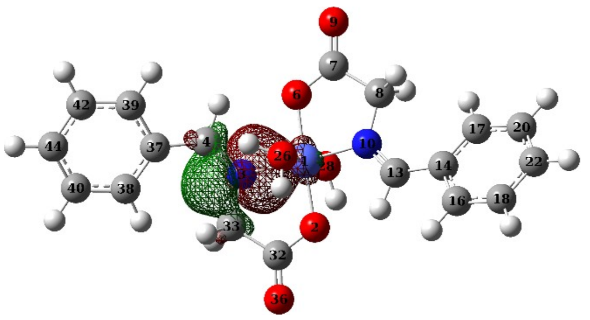

BD(1)Co-N_3	LP(1)N_{10}

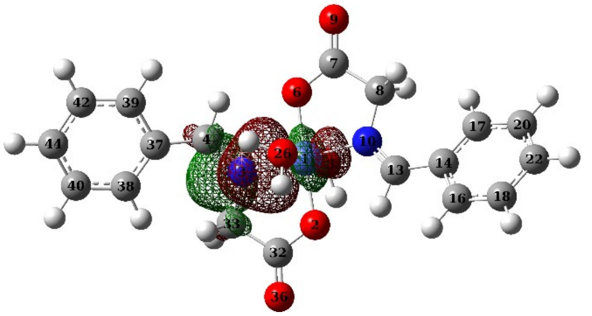

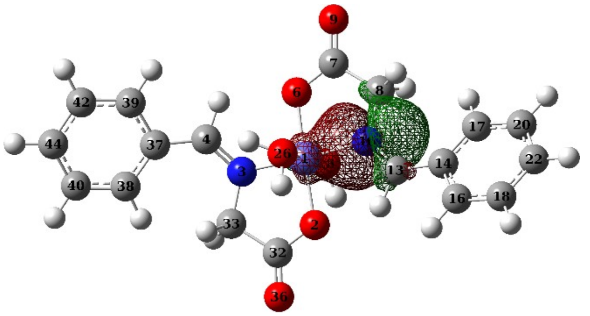

Table S3 Calculated NBOs densities of Cr(III)-L at expected inhibitor-metal interactions.

LP(1)C_{40}	LP(1)Cr
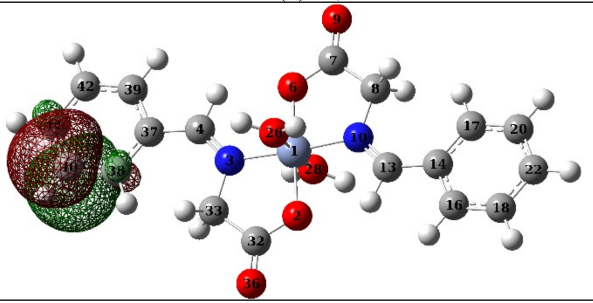	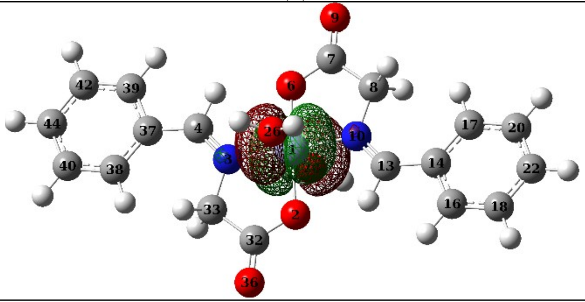
LP(2)O_9	LP(2)O_{36}
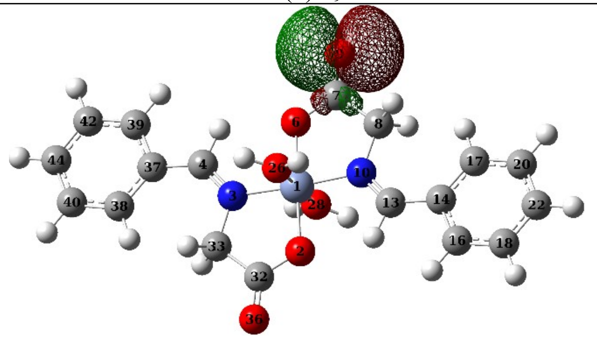	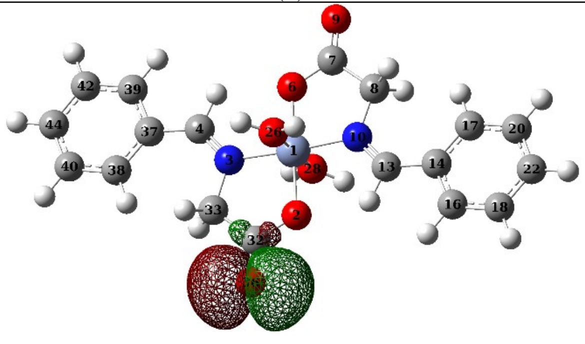
BD(2)C_{18}-C_{22}	BD(2)C_{40}-C_{44}
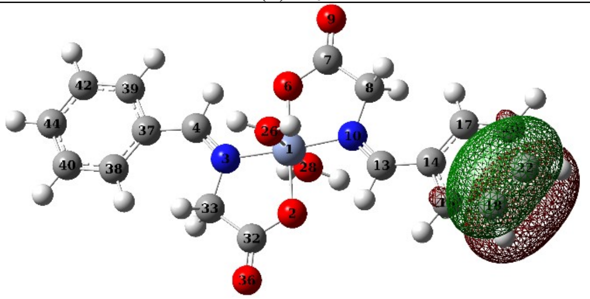	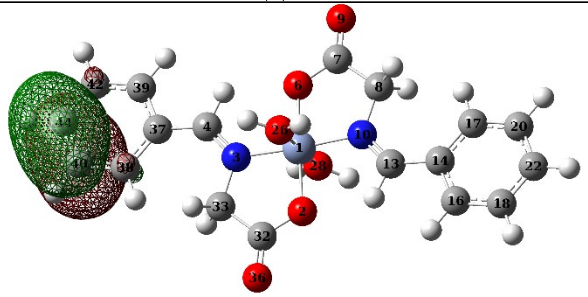
BD(2)C_{14}-C_{16}	BD(2)C_{17}-C_{20}
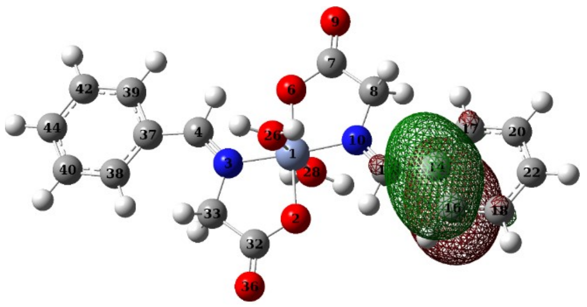	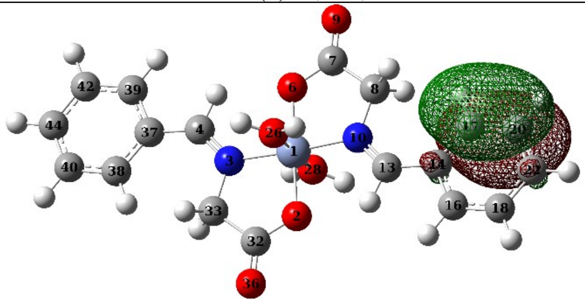
BD(2)C_{37}-C_{38}	LP(1)Cr

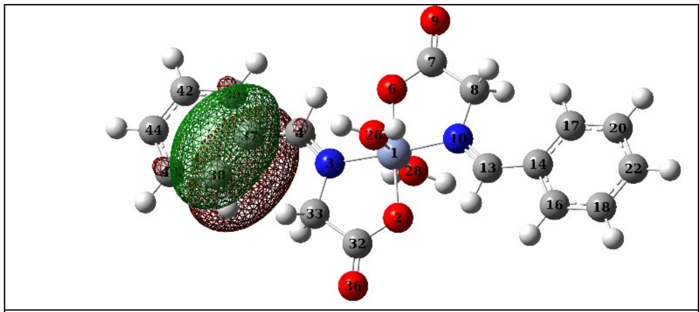	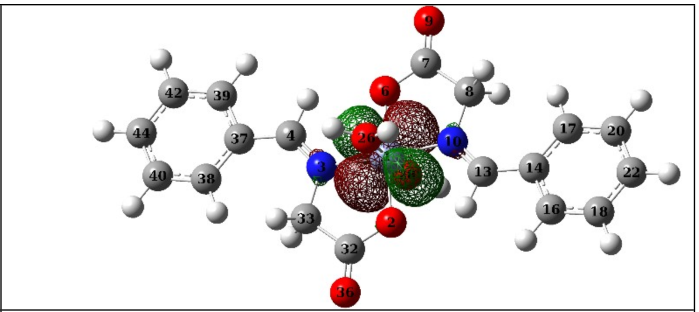
LP(2)O_2	LP(2)O_6
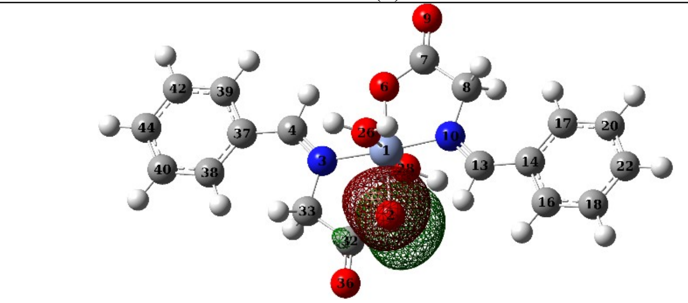	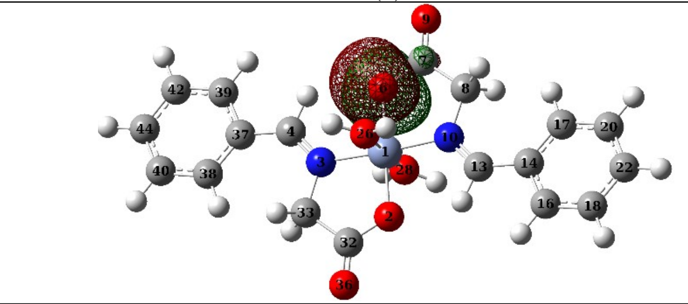
BD(2)C_7-O_9	BD(2)C_{32}-O_{36}
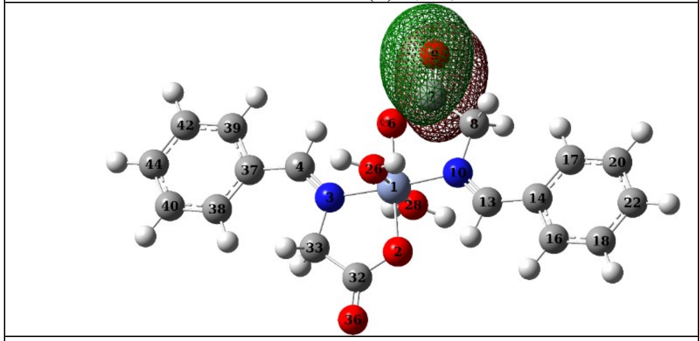	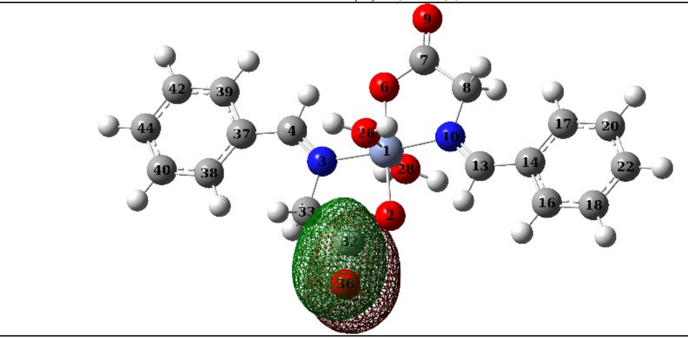
BD(2)N_{10}-C_{13}	BD(2)N_3-C_4
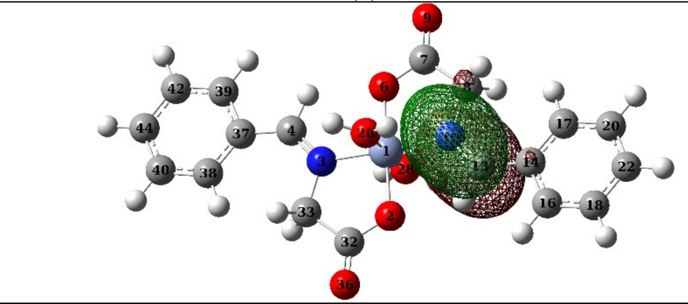	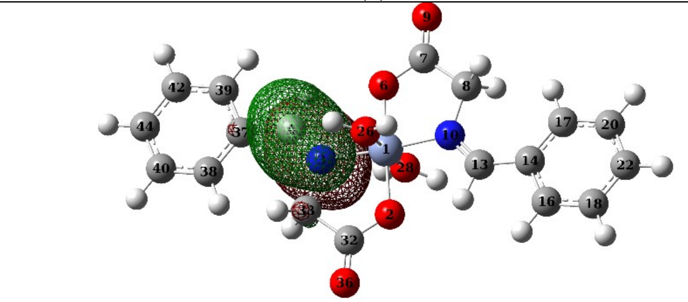
LP(1)O_{28}	LP(1)O_{26}
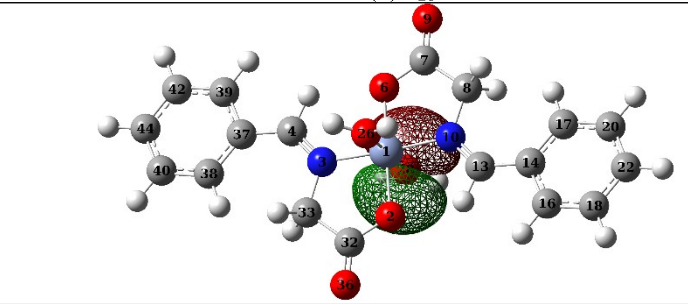	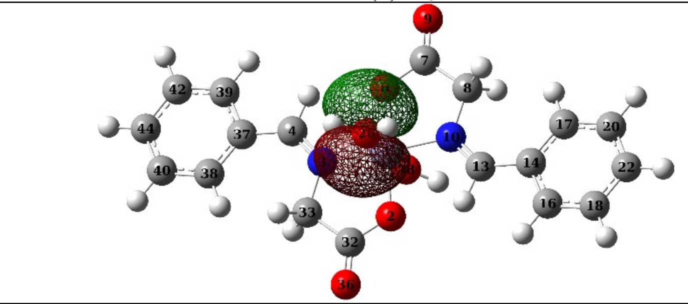
LP(1)N_3	LP(1)N_{10}

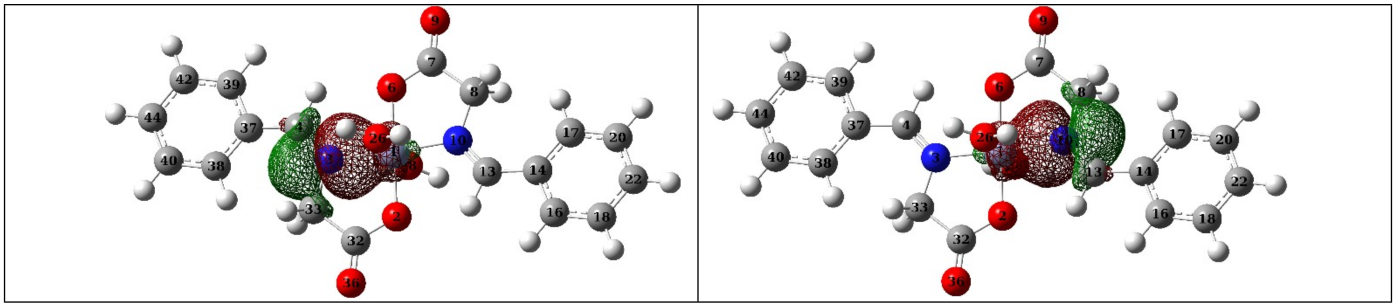

Table S4 Condensed Fukui functions of ligand molecule.

Atoms	Charges[a]	f^+	f^-	Δf^b
N_8	-0.189	0.085	0.114	0.029
C_7	0.056	0.042	0.109	0.067
C_1	-0.039	0.106	0.098	-0.008
C_5	-0.032	0.058	0.061	0.003
C_2	-0.047	0.062	0.056	-0.006
C_3	-0.042	0.051	0.054	0.003
C_4	-0.008	0.076	0.051	-0.025
O_{11}	-0.298	0.101	0.050	-0.051
C_6	-0.043	0.047	0.049	0.002
C_9	-0.001	0.027	0.026	-0.001
C_{10}	0.232	0.032	0.019	-0.013
O_{12}	-0.191	0.031	0.007	-0.024

[a]Hirshfeld charges at B3LYP/6-31G(d,p).

[b]$\Delta f = f^- - f^+$.
